# Cost-effectiveness of financial incentives for improving diet and health through Medicare and Medicaid: A microsimulation study

**DOI:** 10.1371/journal.pmed.1002761

**Published:** 2019-03-19

**Authors:** Yujin Lee, Dariush Mozaffarian, Stephen Sy, Yue Huang, Junxiu Liu, Parke E. Wilde, Shafika Abrahams-Gessel, Thiago de Souza Veiga Jardim, Thomas A. Gaziano, Renata Micha

**Affiliations:** 1 Friedman School of Nutrition Science and Policy, Tufts University, Boston, Massachusetts, United States of America; 2 Brigham and Women’s Hospital, Boston, Massachusetts, United States of America; 3 Harvard T.H. Chan School of Public Health, Boston, Massachusetts, United States of America; Centers for Disease Control and Prevention, UNITED STATES

## Abstract

**Background:**

Economic incentives through health insurance may promote healthier behaviors. Little is known about health and economic impacts of incentivizing diet, a leading risk factor for diabetes and cardiovascular disease (CVD), through Medicare and Medicaid.

**Methods and findings:**

A validated microsimulation model (CVD-PREDICT) estimated CVD and diabetes cases prevented, quality-adjusted life years (QALYs), health-related costs (formal healthcare, informal healthcare, and lost-productivity costs), and incremental cost-effectiveness ratios (ICERs) of two policy scenarios for adults within Medicare and Medicaid, compared to a base case of no new intervention: (1) 30% subsidy on fruits and vegetables (“F&V incentive”) and (2) 30% subsidy on broader healthful foods including F&V, whole grains, nuts/seeds, seafood, and plant oils (“healthy food incentive”). Inputs included national demographic and dietary data from the National Health and Nutrition Examination Survey (NHANES) 2009–2014, policy effects and diet-disease effects from meta-analyses, and policy and health-related costs from established sources. Overall, 82 million adults (35–80 years old) were on Medicare and/or Medicaid. The mean (SD) age was 68.1 (11.4) years, 56.2% were female, and 25.5% were non-whites. Health and cost impacts were simulated over the lifetime of current Medicare and Medicaid participants (average simulated years = 18.3 years). The F&V incentive was estimated to prevent 1.93 million CVD events, gain 4.64 million QALYs, and save $39.7 billion in formal healthcare costs. For the healthy food incentive, corresponding gains were 3.28 million CVD and 0.12 million diabetes cases prevented, 8.40 million QALYs gained, and $100.2 billion in formal healthcare costs saved, respectively. From a healthcare perspective, both scenarios were cost-effective at 5 years and beyond, with lifetime ICERs of $18,184/QALY (F&V incentive) and $13,194/QALY (healthy food incentive). From a societal perspective including informal healthcare costs and lost productivity, respective ICERs were $14,576/QALY and $9,497/QALY. Results were robust in probabilistic sensitivity analyses and a range of one-way sensitivity and subgroup analyses, including by different durations of the intervention (5, 10, and 20 years and lifetime), food subsidy levels (20%, 50%), insurance groups (Medicare, Medicaid, and dual-eligible), and beneficiary characteristics within each insurance group (age, race/ethnicity, education, income, and Supplemental Nutrition Assistant Program [SNAP] status). Simulation studies such as this one provide quantitative estimates of benefits and uncertainty but cannot directly prove health and economic impacts.

**Conclusions:**

Economic incentives for healthier foods through Medicare and Medicaid could generate substantial health gains and be highly cost-effective.

## Abstract 2

Yujin Lee and colleagues model for the health benefits and cost-effectiveness of providing subsidies on fresh fruit and vegetables and other healthy foods via Medicaid and Medicare.

## Author summary

**Why was this study done?:**

- In nearly all nations, healthcare spending continues to increase dramatically, with diet-related conditions being a major driver.
- Fruit and vegetable (F&V) prescriptions and other incentives for healthier eating have been implemented and proposed in healthcare systems but not at scale.
- Thus, the health impacts, costs, and cost-effectiveness of healthy food prescriptions in healthcare are not well established.

**What did the researchers do and find?:**

- As a part of the Food Policy Review and Intervention Cost-Effectiveness (Food-PRICE) Project (www.food-price.org), we estimated the health and economic impacts of healthy food prescriptions in Medicare and Medicaid, the two largest United States federal health insurance programs, which together cover 1 in 3 US citizens.
- Using nationally representative data and a validated microsimulation model (CVD-PREDICT), we evaluated two policy scenarios for adults in Medicare and Medicaid: (1) a 30% subsidy on F&V (F&V incentive) and (2) a 30% subsidy on several healthful foods including F&V, whole grains, nuts/seeds, seafood, and plant oils (healthy food incentive).
- Over a lifetime, the F&V incentive would prevent 1.93 million cardiovascular disease (CVD) events and 0.35 million CVD deaths and save $40 billion in healthcare costs. The healthy food incentive would prevent 3.28 million CVD cases, 0.62 million CVD deaths, and 0.12 million diabetes cases and save $100 billion in healthcare costs.
- Both programs were highly cost-effective from a healthcare perspective—with lifetime incremental cost-effectiveness ratios (ICERs) of $18,184 per quality-adjusted life year (QALY) for the F&V incentive and $13,194/QALY for the healthy food incentive—and from a societal perspective (ICER: $14,576/QALY and $9,497/QALY, respectively).
- Results were consistent across subgroups within each insurance group including by age, race/ethnicity, education, income, and participation status in the Supplemental Nutrition Assistant Program (SNAP).

**What do these findings mean?:**

- Implementing healthy food prescriptions within large government healthcare programs to promote healthier eating could generate substantial health gains and be highly cost-effective.
- Our findings support the implementation and evaluation of such programs within private and public healthcare systems.
- In the US, a new $25 million Produce Prescription Program was just passed in the 2018 Farm Bill, which will provide funding support for such pilot projects over the next 5 years.

## Introduction

US healthcare expenditures have tripled over 50 years, from 5% of gross domestic product in 1960 to 17.9% in 2016 [1]. Medicare and Medicaid are the nation’s two largest public insurance programs [2], serving adults aged 65+ years, people with disabilities, and low-income populations. About 1 in 3 US citizens are covered by these insurance programs, which represent about 25% of the entire federal budget [3]. Key health outcomes remain suboptimal [4] and costs continue to rise [5], highlighting the need for new, cost-effective approaches.

Direct economic incentives to patients have been proposed through Medicare or Medicaid to target traditional cardiometabolic risk factors and promote healthier behaviors, including weight loss, cholesterol, blood pressure control [6,7], and also—now in the 2018 Farm Bill—healthier eating [8]. Suboptimal diet is a leading risk factor for cardiometabolic diseases including coronary heart disease (CHD), stroke, and type 2 diabetes [9,10] and has been linked to nearly half of all estimated annual cardiometabolic deaths in the US [11]. Innovative healthcare strategies for better eating, such as fruit and vegetable (F&V) prescriptions and subsidies [8,12], hold promise to reduce economic and health burdens from cardiometabolic diseases. For example, the 2018 Farm Bill includes $25 million for a new Produce Prescription Program to test healthcare interventions of financial or nonfinancial patient incentives to increase F&V intake. Although research suggests healthy food subsidies are cost-effective outside of healthcare [13–15], the potential impacts on cardiometabolic health, healthcare costs, and cost-effectiveness within healthcare are not well established.

To address these gaps in knowledge and inform health policy and future interventional projects, we used a validated microsimulation model to estimate the potential cardiometabolic health and economic impacts of a financial incentive program for healthful foods in Medicare and Medicaid adult beneficiaries. This investigation was performed as a part of the Food Policy Review and Intervention Cost-Effectiveness (Food-PRICE) Project (www.food-price.org).

## Methods

### Study overview

The potential health and economic impacts of implementing a healthful food incentive program through Medicare and Medicaid were modeled using the validated CVD-PREDICT microsimulation model [16] in closed cohorts over 5, 10, and 20 years (2018–2037) and over the lifetime of current participants. We obtained baseline characteristics, risks, dietary habits, and disease incidence data from the National Health and Nutrition Examination Surveys (NHANES) 2009 through 2014 cycles. The model utilized the national data to assess cumulative cardiometabolic health outcomes and costs based on current trends as well as alternative scenarios of specific interventions. At each stage of the logic pathway (S1 Appendix Fig A), the best available evidence, supplemented with reasoned assumptions (S1 Appendix Table A), was used to estimate the potential health and economic impacts of the proposed incentive program in Medicare and Medicaid adult beneficiaries. The analysis plan is presented in S1 Appendix Text A. Model inputs, sources, and key assumptions are described in detail below (Table 1).

**Table 1:** Key model inputs and sources for cost-effectiveness analysis of financial incentives for improving diet and health through Medicare and Medicaid using the CVD-PREDICT model^a^.

Model inputs	Value	Source
Baseline characteristics^b^		
Baseline demographics	S1 Appendix Table K	NHANES 2009–2014[17]
Baseline CVD risk factors		
Baseline prevalent disease		
Baseline dietary intakes		
Policy effects^c^	S1 Appendix Table C	Afshin 2017 [18] Green 2013 [19]
Price elasticity for intake of healthful foods for low income (PIR < 1.3) per 30% decrease in price, %	40.5	
Price elasticity for intake of healthful foods for high income (PIR ≥ 1.3) per 30% decrease in price, %	34.3	
Diet-disease etiologic effects^d^	S1 Appendix Table D	Micha 2017 [11]
CHD		
Fruits, per 100 g/day	0.93 (0.89, 0.97)	
Vegetables, per 100 g/day	0.94 (0.91, 0.97)	
Nuts/seeds, per 1 oz (28 g)/week	0.91 (0.87, 0.94)	
Whole grains, per 50 g/day	0.96 (0.93, 0.99)	
Seafood ω-3 fats, per 100 mg/day	0.82 (0.75, 0.90)	
PUFA replacing carbs, per 5% energy/day	0.88 (0.83, 0.94)	
Ischemic stroke		
Fruits, per 100 g/day	0.86 (0.80, 0.92)	
Vegetables, per 100 g/day	0.80 (0.70, 0.92)	
Whole grains, per 50 g/day	0.90 (0.83, 0.97)	
Hemorrhagic stroke		
Fruits, per 100 g/day	0.69 (0.56, 0.84)	
Vegetables, per 100 g/day	0.80 (0.67, 0.96)	
Whole grains, per 50 g/day	0.90 (0.83, 0.97)	
Type 2 diabetes		
Nuts/seeds, per 1 oz (28 g)/week	0.96 (0.94, 0.98)	
Whole grains, per 50 g/day	0.86 (0.80, 0.92)	
Policy costs^e^	S1 Appendix Table H	
Administrative costs, % of total subsidy costs	5–20	SNAP [20], CMS [21]
Subsidy costs		USDA ERS Quarterly Food-at-Home Price Database [22]
Fruits, per 100 g	$0.34	
Vegetables, per 100 g	$0.29	
Nuts/seeds, per 100 g	$0.76	
Whole grains, per 100 g	$0.64	
Seafood, per 100 g	$1.15	
Plant oils, per 100 g	$0.76	
Health-related costs^f^	S1 Appendix Table I	
Formal healthcare costs		
CVD costs		
Chronic disease states, per year	$2,222–$3,362	Lee 2010 [23], Pignone 2006 [24]
Acute disease states, per year	$20,092–$58,254	O’Sullivan 2011 [25]
Procedures and repeat events	$20,092–$58,254	O’Sullivan 2011 [25]
Screening	$1–$79	Pletcher 2009 [26], Lazar 2011 [27]
Medications, per year	$8–$280	Redbook 2009 [28], Nuckols 2011 [29], Pignone 2006 [24], Shah 2011 [30]
Statin-associated adverse events	$185–$7,280	Lee 2010 [23]
Diabetes costs		ADA 2013 [31], Zhuo 2013 [32]
Institutional care, per year	$1–$2,495	
Outpatient care, per year	$7–$501	
Medications and supplies, per year	$35–$1,043	
Informal healthcare costs		
Time per outpatient visit, per minute		Russell 2008 [33]
Travel	35	
Waiting	42	
Wage for adults aged >45 years, per hour	$15.19	Bureau of Labor Statistics 2013 [34]
Productivity costs, dollars		Kim 2016 [35]
Labor force participation rates as full-time workers by age group	0.076–0.845	
Average annual earnings by age group, per year	$38,723–$55,363	

^a^ All costs inflated to constant 2017 dollars using the Bureau of Labor Statistics’ Consumer Price Index [36].

^b^ Details are presented in S1 Appendix Table K.

^c^ Details are presented in S1 Appendix Table C. Low-income individuals as defined by their income eligibility threshold for government food-assistance programs (PIR of 1.3).

^d^ Details are presented in S1 Appendix Table D. Values represent RRs for increased consumption of each dietary factor and cardiometabolic disease risk at age 50 (45–54 years). RRs for other age groups are presented in S1 Appendix Table D.

^e^ Details are presented in S1 Appendix Table H.

^f^ The ranges represent multiple sub-cost values under each cost category. Details are presented in S1 Appendix Table I.

Abbreviations: ADA, American Diabetes Association CHD, coronary heart disease; CMS, Centers for Medicare and Medicaid Service; CVD, cardiovascular disease; ERS, Economic Research Service; NHANES, National Health and Nutrition Examination Survey; oz, ounce; PIR, poverty–income ratio; PUFA, polyunsaturated fatty acids; RR, relative risk; SNAP, Supplemental Nutrition Assist Program; USDA, US Department of Agriculture.

### Medicare/Medicaid policy scenarios

We modeled two distinct interventions within the Medicare/Medicaid program, compared with a base case of no new intervention: (1) 30% subsidy on F&V (F&V incentive) and (2) 30% subsidy on broader healthful foods (healthy food incentive) including F&V, whole grains, nuts/seeds, seafood, and plant oils (see S1 Appendix Table B for dietary target details and definitions). A secondary analysis was performed excluding incentives for seafood and plant oils, the two most expensive food categories, from the healthy food incentive. The subsidy level of 30% was based on the subsidy level used in the US Department of Agriculture’s (USDA’s) Healthy Incentives Pilot (HIP), a randomized controlled trial implemented among Supplemental Nutrition Assistant Program (SNAP) participants to incentivize F&V consumption [37].

We modeled the implementation of each intervention through adaptation of the existing electronic benefits transfer (EBT) system, currently used for federal food-assistance programs [38], for use in Medicaid and Medicare. Each beneficiary would receive an EBT card linked to product-identifying universal product codes, subsidizing 30% of purchases of targeted foods at point of purchase across diverse retail locations already accepting EBT. For example, for every dollar spent on targeted healthful foods, $0.30 would be paid by the EBT card, with the remaining $0.70 covered out of pocket.

### Simulated US population

Our population was based on US adults aged 35–80 years at baseline across three cycles of NHANES (2009–2014) and enrolled in Medicare and/or Medicaid, defined by reporting Medicare and/or Medicaid insurance coverage in the health insurance questionnaire. The sociodemographic characteristics and cardiometabolic risk factors of these participants were derived from NHANES, including dietary habits from two 24-hour recalls as previously described [39]. A simulated nationally representative Medicare, Medicaid, and dual-eligible population of 1,000,000 individuals was generated by weighted sampling with replacement using NHANES survey weights to account for the complex survey design and sampling [40]. All individuals were simulated until death or age 100 years, whichever came first.

### Policy effects on dietary intakes

Details of the policy effects and corresponding calculations are provided in S1 Appendix Table C. Estimated policy effects accounted for the expected change in consumption of each healthful food in response to a 30% price change, differential price responsiveness by income status, and the percentage of healthful food purchased at retail locations that would accept the EBT card (e.g., supermarkets and grocery stores, as opposed to restaurants, cafeterias, and food banks). Consistent with findings of studies using price changes to alter dietary behaviors [18], we assumed the time lag between policy implementation and dietary changes occurred within 1 year, with the intervention effect sustained throughout the subsidized period.

The effect of the price change on dietary intakes was derived from a systematic review and meta-analysis of interventional and prospective observational studies of changes in food price in relation to dietary consumption [18]. This meta-analysis demonstrated that each 1% change in price results in a 1.24% change in intake of healthful foods. To account for differential price responsiveness by socioeconomic status, we incorporated a proportional 18.2% higher price responsiveness for lower- versus higher-income individuals (utilizing the family income eligibility threshold for government food-assistance programs, i.e., a poverty–income ratio [PIR] of 1.3), based on a meta-analysis that compared price responses between lower- and higher-income households within high-income countries [19]. We recognized that these estimates represented the average policy effect in the population, accounting for higher or lower effectiveness across different individuals. Of note, the resulting estimated effects on F&V intake for a 30% subsidy based on these two meta-analyses (for PIR ≥ 1.3, 30.9% for fruits and 23.7% for vegetables; and for PIR < 1.3, 35.2% and 28.7%, respectively) were comparable to findings from the HIP trial (26% effect on combined F&V) [37].

### Effects of dietary changes on cardiometabolic risk

Our detailed methods for reviewing and synthesizing the evidence to estimate effect sizes for associations between dietary factors and cardiometabolic endpoints have been reported [9,11]. Meta-analyses of prospective cohort studies or randomized controlled trials provided estimates for direct effects of each dietary factor on CHD, stroke, or type 2 diabetes, including associations of F&V with CHD and stroke; whole grains with CHD, stroke, and type 2 diabetes; nuts/seeds with CHD and diabetes; and polyunsaturated and seafood ω-3 fats with CHD (S1 Appendix Table D). A declining proportional effect by age was incorporated, based on age patterns of cardiometabolic risk factors and clinical events [9,11]. Because these observed etiologic effects assess how dietary differences relate to clinical risk in free-living populations, they inherently incorporate the overall impact due to average dietary complements and substitutes in the population. We have published detailed validity analyses evaluating the extent to which these estimated etiologic effects of dietary factors may be biased by confounding or measurement error [9,11]. These included comparing estimated etiologic effects for individual dietary components to (1) observed associations of overall dietary patterns with clinical endpoints in long-term cohorts, (2) effects of dietary patterns on low-density lipoprotein (LDL) cholesterol and systolic blood pressure in randomized controlled feeding trials, and (3) effects of dietary patterns on clinical cardiovascular disease (CVD) endpoints in a large controlled clinical trial (S1 Appendix Text B; Tables E-G). Each of these analyses demonstrated that the estimated etiologic effects from individual dietary components were very similar to what would be expected based on these other lines of evidence.

### Microsimulation model structure and outputs

**Fig 1:**
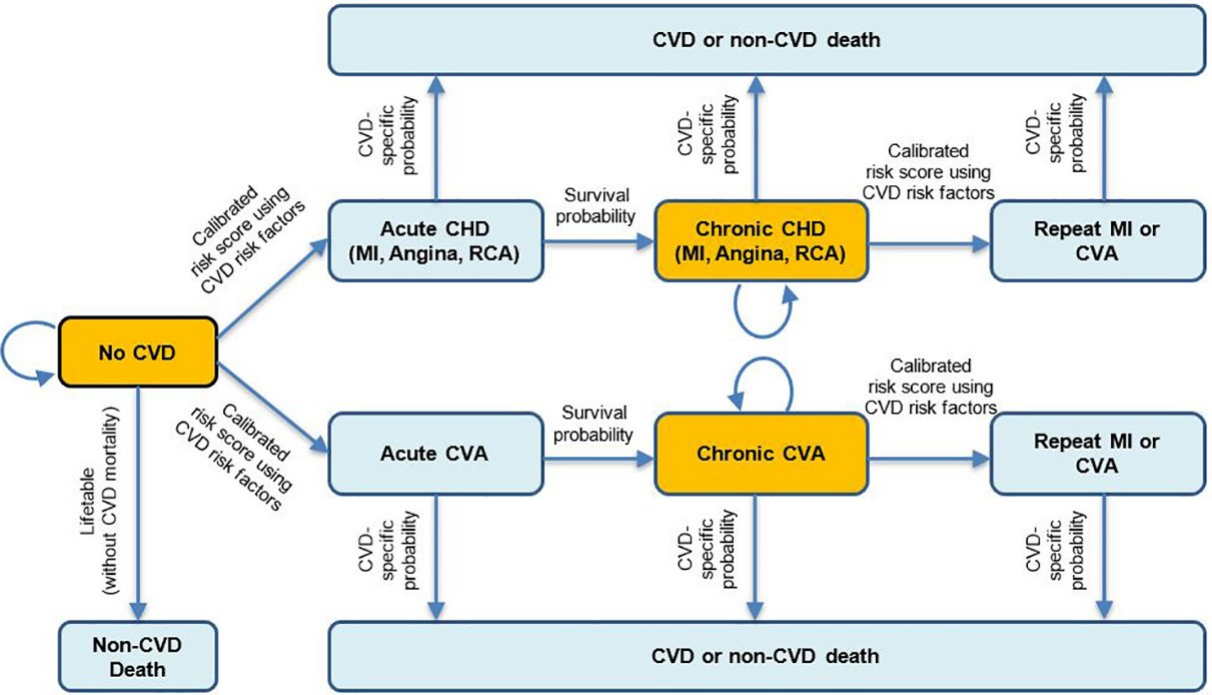
The CVD-PREDICT microsimulation model. Transitions were based on a calibrated risk score including age, sex, systolic blood pressure, total cholesterol, HDL cholesterol, current smoking, and diabetes status. Baseline risk factors were derived from NHANES 2009–2014, with further annual changes in all risk factors incorporating both age and secular trends. Increased intake of healthful foods could decrease the probability of transitioning of no CVD to acute CVD and chronic CVD to recurring CVD or CVD death. *Adapted from Mozaffarian D*, *et al (2018) Cost-effectiveness of financial incentives and disincentives for improving food purchases and health through the US Supplemental Nutrition Assistance Program (SNAP)*: *A microsimulation study*. *https*:*//doi*.*org/10*.*1371/journal*.*pmed*.*1002661*. CHD, coronary heart disease; CVA, cerebrovascular accident; CVD, cardiovascular disease; HDL, high-density lipoprotein; MI, myocardial infarction; NHANES, National Health and Nutrition Examination Survey; RCA, resuscitated cardiac arrest.

CVD-PREDICT is a validated discrete time microsimulation model coded in C++ that is used to simulate and quantify effects of policies on CHD, stroke, and diabetes, with annual updating of each health state (S1 Appendix Text C) [41,42]. The model is run at the level of individuals, which incorporates the probability of their health transition based on each person’s risk factors. Using data from NHANES 2009–2014, the model was populated with simulated individuals on Medicare and/or Medicaid including their risk factors such as age, sex, systolic blood pressure, total cholesterol, high-density lipoprotein (HDL) cholesterol, smoking, diabetes, and current dietary habits. Other model variables included validated CHD and stroke risk equations and case fatality risks based on a calibrated Framingham-based risk function as well as validated empiric historical disease trends [43]. CVD risk factors, the subsequent estimated CVD incidence and mortality, and diabetes incidence were extrapolated and updated using age and time trends from NHANES. At any given time point, a simulated individual could be in one health state, with the probability of experiencing subsequent events based on individual’s cardiometabolic risk factors and dietary habits. The microsimulation process across each state and transitions is shown in Fig 1.

Model outputs included total CVD events, CVD deaths, and diabetes cases at 5, 10, and 20 years and cohort lifetime. The specific model outcomes included deaths from CHD or stroke; nonfatal events including myocardial infarction, stroke, angina, resuscitated cardiac arrest, and diabetes incidence; quality-adjusted life years (QALYs); and event-associated health-related costs. Outputs were estimated for overall Medicare and Medicaid population and further by insurance group (Medicare, Medicaid, dual-eligible). To investigate consistency of health and economic impacts of each program across subgroups within each insurance group, analyses were further stratified by age (35–64, ≥65 years), race/ethnicity (non-Hispanic white, non-Hispanic black, Hispanic, other), education (<high school, high school or some college, college graduate or above), SNAP (SNAP participants, SNAP-eligible nonparticipants, SNAP-ineligible individuals), and income (PIR < 1.3 or ≥ 1.3) within Medicare; by race/ethnicity, education, SNAP, and age (35–54, 55–74, ≥75 years) within Medicaid; and by race/ethnicity, education, and SNAP within dual-eligible participants. Income strata are not shown within Medicaid and dual-eligible because beneficiaries are of low income.

### Policy and health-related costs

Policy costs included the administrative costs of program implementation and the subsidy costs for incentivized foods (S1 Appendix Table H, Text D). Estimated administrative costs included costs of personnel, training, use of the EBT system, and monitoring and evaluation, which were derived from reports from SNAP [20] and the Centers for Medicare and Medicaid Services (CMS) [21]. Administrative costs were assumed to be higher (20% of total subsidy costs) in the first year of implementation, based on SNAP administrative costs in 2004 when the EBT system was implemented in all states [20], and lower (5% of total subsidy costs) in subsequent years, based on CMS data demonstrating overall administrative costs of Medicaid to be about 5% of total expenditures [21]. The costs of food incentives were calculated for each food category based on data from the USDA Economic Research Service Quarterly Food-at-Home Price Database [22].

Health-related costs included formal healthcare, informal healthcare, and productivity costs (S1 Appendix Table I). Formal healthcare costs included for CVD all acute and chronic disease states, surgical procedures, screening, treatments, and statin-associated side effects; for diabetes, costs included institutional care, outpatient care, outpatient medications, and supplies. Informal healthcare costs included costs for patients’ travel and waiting time as derived from the Bureau of Labor Statistics [34]. Productivity costs were calculated using age-specific average annual earnings derived from the Current Population Survey [44].

### Cost-effectiveness analysis

We followed recommendations from the Second Panel on Cost-Effectiveness in Health and Medicine [45]. Analyses were conducted from two perspectives: (1) healthcare perspective, incorporating policy costs and formal healthcare costs, and (2) societal perspective, further incorporating informal healthcare and productivity costs. All costs were inflated to 2017 US dollars using the Consumer Price Index [46], and all costs and QALYs were discounted at 3% annually. Net costs were calculated as policy costs minus health-related cost savings. Incremental cost-effectiveness ratios (ICERs) were calculated as the net change in costs divided by the net change in QALYs. Willingness-to-pay thresholds were evaluated at $150,000 and $50,000 per QALY, as recommended by the American College of Cardiology and American Heart Association [47].

### Sensitivity and uncertainty analyses

To assess the potential impact of uncertainty in key model inputs, probabilistic sensitivity analyses incorporated the uncertainty distributions of multiple variables including policy effect sizes, diet-disease relative risks (including their variation by age), food costs, formal healthcare costs, and utility weights (S1 Appendix Table J). Drawing from the uncertainty distributions of each of these inputs, 1,000 simulations were run at 5 years and over a lifetime, with 95% uncertainty intervals based on the 2.5th and 97.5th percentiles of the 1,000 simulations. One-way sensitivity analyses were also evaluated to test the health and economic impacts of lower (20%) and higher (50%) food subsidy levels for the two interventions.

## Results

### Baseline characteristics and dietary intakes

Medicare beneficiaries were more likely to be older and white and have higher incomes compared with other insurance groups (S1 Appendix Table K). Adults on Medicaid were younger, but other demographics were more similar to dual-eligible beneficiaries. Health characteristics were generally similar across insurance groups. Baseline consumption of targeted healthful foods was higher among individuals on Medicare compared with Medicaid or dual-eligible.

Compared to no new intervention, the F&V incentive would increase mean intakes of fruits by 41.2 g/day (approximately 0.4 servings/day) and vegetables by 43.9 g/day (approximately 0.4 servings/day) (S1 Appendix Table L). The healthy food incentive would similarly increase intakes of F&V and further increase mean intakes of whole grains by 8.1 g/day (approximately 0.2 servings/day), nuts/seeds by 3.8 g/day (approximately 0.1 servings/day), seafood by 4.8 g/day (approximately 0.2 servings/day), and plant oils by 6.0 g/day (approximately 1.5 teaspoon) in the overall combined population.

### Health outcomes

**Fig 2:**
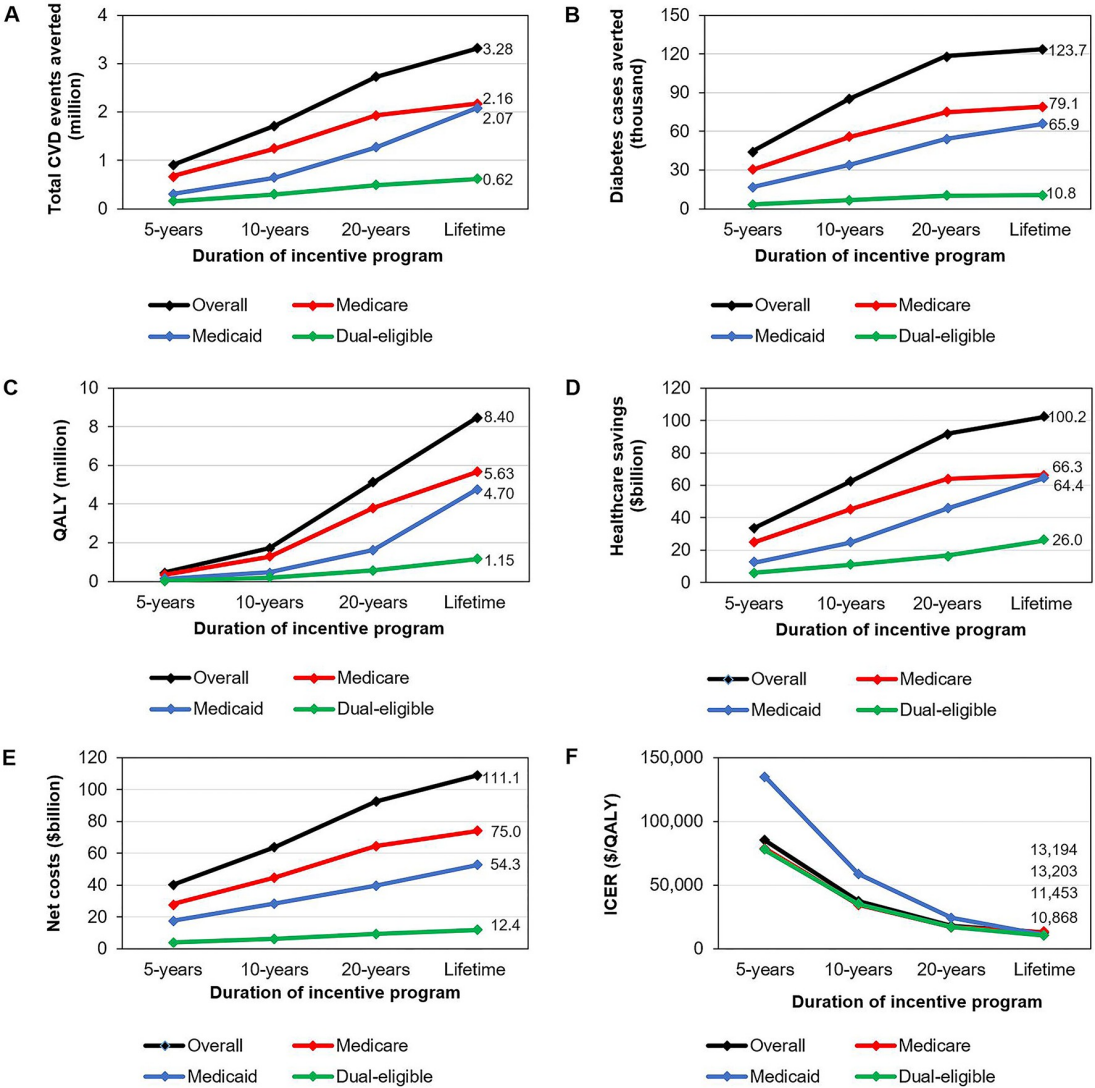
**Estimated reductions in total (A) CVD events averted, (B) diabetes cases averted, (C) QALYs, (D) healthcare savings, (E) net costs, and (F) ICER of the 30% healthy food incentive program through Medicare and Medicaid by insurance type over 5, 10, and 20 years and lifetime.** Values are shown from a healthcare perspective. Numbers indicate the values for lifetime analysis. ICERs were calculated as the change in net costs (policy costs minus healthcare savings) divided by the net change in QALYs. CVD, cardiovascular disease; ICER, incremental cost-effectiveness ratio; QALY, quality-adjusted life year.

Over a lifetime, the F&V incentive was estimated to prevent 1.93 million CVD events and 0.35 million CVD deaths and generate 4.64 million QALYs, compared with no new intervention (Table 2). Corresponding values for the healthy food incentive were 3.28 million CVD events and 0.62 million CVD deaths prevented and 8.40 million QALYs gained. This scenario would additionally prevent 0.12 million diabetes cases because of direct links of whole grains and nuts/seeds with diabetes. Absolute health benefits were larger in Medicare because of a larger population size (58.2 million) compared with Medicaid (35.2 million) and dual-eligible (11.4 million). Projected reductions in total CVD events and diabetes cases and projected QALYs gained increased with program duration for both incentive programs (Fig 2, S1 Appendix Fig B).

**Table 2:** Lifetime health gains, costs, and cost-effectiveness of 30% F&V incentive and healthy food incentive programs through Medicare and Medicaid from a healthcare perspective^a^.

	Median Estimate (95% UI)			
	Overall^b^	Medicare^c^	Medicaid^d^	Dual-eligible^e^
US adults (35–80 years old) represented, million	82.0	58.2	35.2	11.4
Scenario 1: F&V incentive (30%)				
Cases averted, million				
CVD events	1.93 (1.57, 2.31)	1.29 (1.01, 1.56)	1.15 (0.95, 1.41)	0.37 (0.30, 0.44)
CVD deaths	0.35 (0.28, 0.42)	0.25 (0.20, 0.30)	0.15 (0.12, 0.18)	0.04 (0.03, 0.05)
Diabetes cases^f^	−0.006 (−0.008, −0.005)	−0.003 (−0.005, −0.002)	−0.010 (−0.012, −0.009)	−0.0034 (−0.0038, −0.0031)
QALYs gained, million^g^	4.64 (3.69, 5.69)	3.20 (2.46, 3.95)	2.41 (1.94, 3.02)	0.63 (0.50, 0.79)
Change in policy costs, $ billion^h^				
Administrative costs	7.11 (4.98, 9.81)	4.86 (3.35, 6.65)	3.61 (2.53, 5.01)	1.29 (0.89, 1.77)
Food subsidy costs	115.5 (80.9, 159.5)	78.1 (53.8, 106.8)	61.8 (43.4, 85.8)	21.5 (14.9, 29.7)
Change in formal healthcare cost, $ billion^i^	−39.7 (−48.7, −31.8)	−27.2 (−33.5, −21.0)	−23.0 (−28.2, −18.4)	−9.46 (−11.9, −7.23)
Net costs, $ billion^j^	83.5 (45.2, 129.0)	57.0 (30.6, 87.0)	41.0 (21.0, 64.0)	10.8 (5.3, 17.5)
ICER, $/QALY^k^	18,184 (9,270, 29,371)	17,842 (9,392, 28,998)	16,933 (8,295, 28,007)	17,238 (7,995, 29,407)
Scenario 2: Healthy food incentive (30%)				
Cases averted, million				
CVD events	3.28 (2.87, 3.69)	2.16 (1.84, 2.49)	2.07 (1.82, 2.34)	0.62 (0.53, 0.70)
CVD deaths	0.62 (0.54, 0.71)	0.44 (0.37, 0.50)	0.31 (0.27, 0.35)	0.07 (0.06, 0.08)
Diabetes cases^f^	0.12 (0.10, 0.15)	0.08 (0.06, 0.10)	0.07 (0.05, 0.08)	0.011 (0.007, 0.014)
QALYs gained, million^g^	8.40 (7.23, 9.58)	5.63 (4.81, 6.53)	4.70 (4.11, 5.39)	1.15 (0.98, 1.34)
Change in policy costs, $ billion^h^				
Administrative costs	12.2 (9.61, 15.1)	8.21 (6.51, 10.2)	6.57 (5.21, 8.19)	2.11 (1.66, 2.64)
Food subsidy costs	198.2 (156.3, 245.4)	131.9 (104.6, 163.4)	112.9 (89.6, 140.7)	35.5 (27.8, 44.3)
Change in formal healthcare cost, $ billion^i^	−100.2 (−113.9, −87.0)	−66.3 (−76.3, −57.0)	−64.4 (−73.8, −55.1)	−26.0 (−30.5, −21.5)
Net costs, $ billion^j^	111.1 (67.0, 160.6)	75.0 (44.0, 109.1)	54.3 (28.3, 82.4)	12.4 (5.3, 20.4)
ICER, $/QALY^k^	13,194 (7,741, 19,683)	13,203 (7,454, 19,954)	11,453 (5,662, 17,929)	10,868 (4,527, 18,186)

^a^Health outcomes were evaluated among Medicare, Medicaid, and dual-eligible beneficiaries aged 35–80 years at baseline and followed until death or 100 years of age, whichever came first.

^b^Includes Medicare-only, Medicaid-only, and dual-eligible beneficiaries. The number of overall population (*n* = 82 million) is not equal to sum of Medicare (*n* = 58.2 million) and Medicaid (*n* = 35.2 million) because dual-eligible (*n* = 11.4 million) is included in both Medicare and Medicaid.

^c^Includes Medicare-only and dual-eligible beneficiaries.

^d^Includes Medicaid-only and dual-eligible beneficiaries.

^e^Beneficiaries on both Medicare and Medicaid.

^f^We did not identify probable or convincing evidence of etiologic effects of F&V on diabetes; the F&V incentive resulted in a slightly higher number of diabetes cases compared to a base case of no new intervention because of increased overall survival from prevented CVD.

^g^QALYs were discounted at 3% annually.

^h^Policy costs included total administrative costs and food subsidy costs. All costs were inflated in 2017 dollars.

^i^Negative costs indicate health-related savings. Formal healthcare costs were calculated from the change in total healthcare costs associated with CVD events (including chronic/acute disease states, surgical procedures, screening costs, and drug costs) and with diabetes cases (including institutional care, outpatient care, outpatient medications, and supplies), discounted at 3% annually.

^j^Net costs from a healthcare perspective = Policy costs − formal healthcare savings, discounted at 3% annually.

^k^According to the ACC/AHA, ICERs below $50,000/QALY and at $50,000–$150,000/QALY are considered highly cost-effective and cost-effective, respectively [47].

Abbreviations: ACC/AHA, American College of Cardiology and American Heart Association; CVD, cardiovascular disease; F&V, fruits and vegetables; QALY, quality-adjusted life year; ICER, incremental cost-effectiveness ratio; UI, uncertainty interval.

### Cost-effectiveness

Lifetime policy costs for the F&V incentive and healthy food incentive would be $122.6 billion and $210.4 billion, respectively. From a healthcare perspective, considering policy costs and formal healthcare costs, the F&V incentive was estimated to save $39.7 billion in formal healthcare costs, with net costs of $83.5 billion over a lifetime; corresponding values for the healthy food incentive were larger at $100.2 billion and $111.1 billion, respectively (Table 2). Both scenarios were cost-effective (<$150,000/QALY) at 5 years and highly cost-effective (<$50,000/QALY) at 10 years and beyond, with lifetime ICERs of $18,184/QALY for the F&V incentive and $13,194/QALY for the healthy food incentive (Fig 2, S1 Appendix Fig B). By insurance group, ICERs for the healthy food incentive were substantially higher in Medicaid beneficiaries in the short (5 years) ($135,093/QALY) and medium (10 years) term ($58,888/QALY) compared with Medicare ($78,715/QALY, $34,721/QALY) and dual-eligible ($77,537/QALY, $35,246/QALY). In contrast, lifetime ICERs were modestly higher in Medicare ($13,203/QALY) compared with Medicaid ($11,453/QALY) and dual-eligible ($10,868/QALY) (Fig 2).

From a societal perspective, further including informal healthcare and productivity costs, lifetime net costs were $68.8 billion for the F&V incentive and $80.5 billion for the healthy food incentive (S1 Appendix Table M; Fig C). Lifetime ICERs were $14,576/QALY for the F&V incentive and $9,497/QALY for the healthy food incentive. By insurance group, lifetime ICERs were $10,078/QALY in Medicare, $5,916/QALY in Medicaid, and $5,842/QALY in dual-eligible for the healthy food incentive.

### Probability of cost-effectiveness

**Fig 3:**
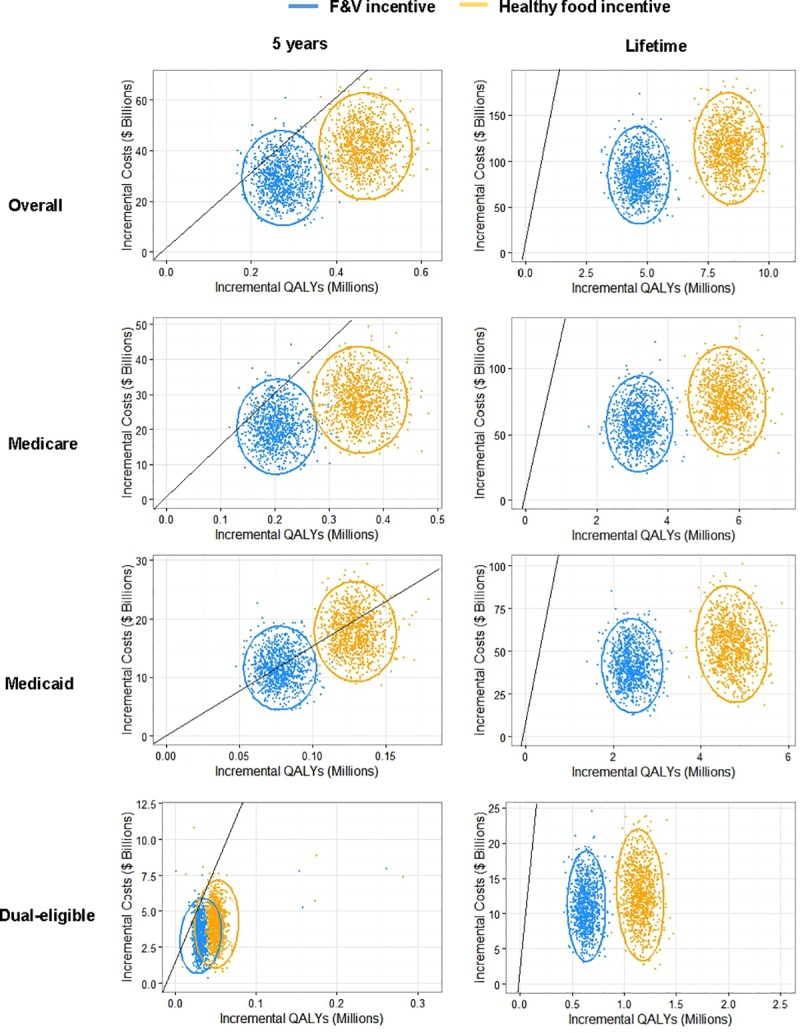
Probabilistic sensitivity analyses for cost-effectiveness of the F&V incentive and healthy food incentive programs through Medicare and Medicaid, by insurance group at 5 years and over a lifetime from a healthcare perspective. Values are presented in cost-effective planes of incremental costs ($ billions) versus incremental QALYs, compared to a base scenario of usual care. For each scenario, each colored dot depicts 1 of 1,000 Monte Carlo iterations, and the ellipse depicts the 95% UIs. Results are presented from the healthcare perspective. The solid black lines represent a value of $150,000/QALY, a recommended threshold for assessing health interventions, with values to the right of the line being cost-effective with an ICER < $150,000/QALY. Note: Because of the different population sizes of each beneficiary group, different axis scales were utilized for each panel. F&V, fruits and vegetables; ICER, incremental cost-effectiveness ratio; QALY, quality-adjusted life year; UI, uncertainty interval.

At 5 years, the probabilities that the F&V incentive would be cost-effective were 0.886, 0.909, 0.506, and 0.859 for overall, Medicare, Medicaid, and dual-eligible beneficiaries, respectively, whereas for the healthy food incentive corresponding values were 0.997, 0.999, 0.667, and 0.98, respectively (Fig 3, S1 Appendix Table N). Over a lifetime, the probability of both incentive programs being cost-effective was 1.00 (1,000 of 1,000 simulations) for overall, Medicare, Medicaid, and dual-eligible beneficiaries.

### Subgroup and one-way sensitivity analyses

**Fig 4:**
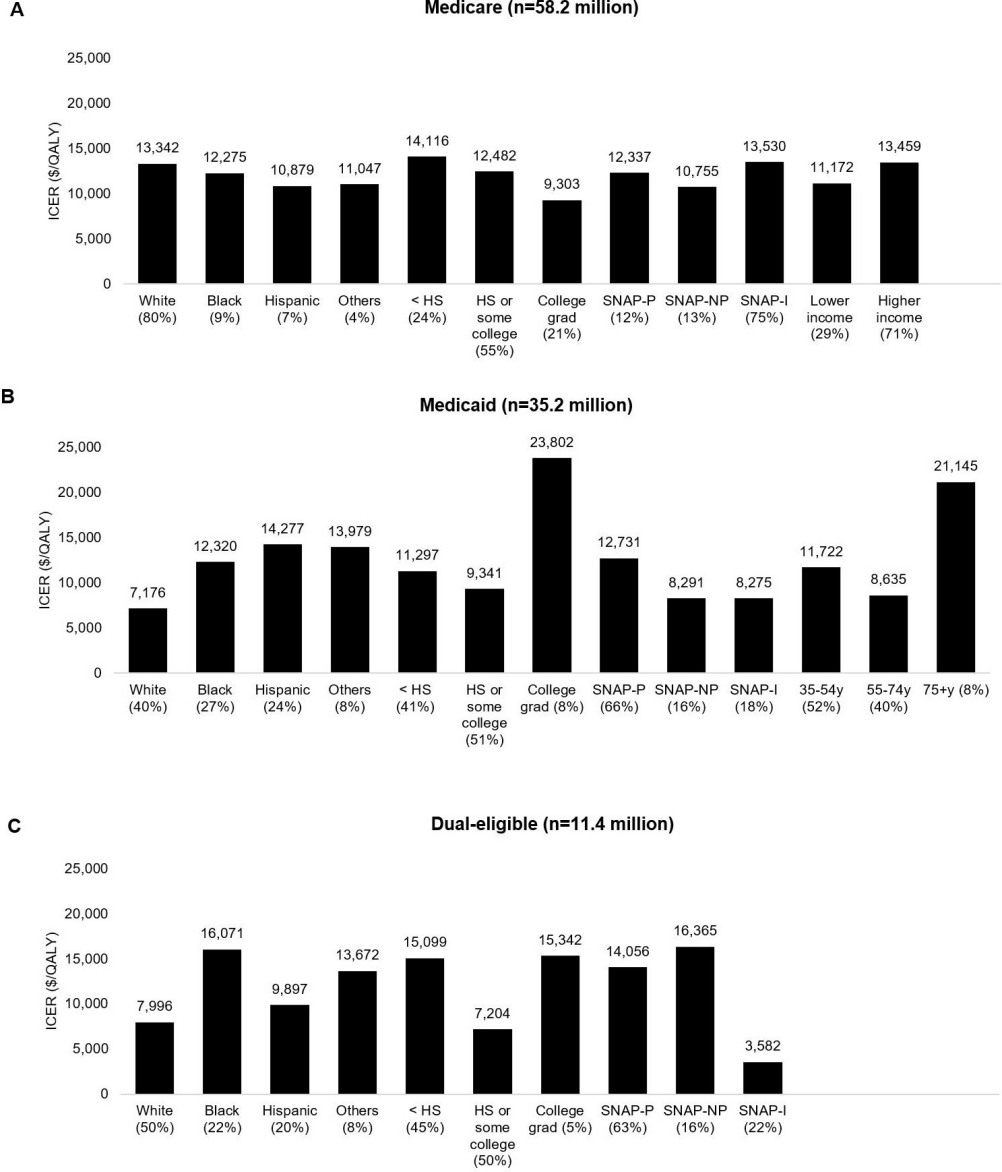
**Lifetime cost-effectiveness of the 30% healthy food incentive program in (A) Medicare, (B) Medicaid, and (C) dual-eligible adult beneficiaries by race/ethnicity, education, SNAP, income, and age.** Values are shown from a healthcare perspective. Numbers represent ICERs. ICERs were calculated as the change in net costs (policy costs minus health-related savings) divided by the net change in QALYs. Stratified analyses were conducted by race/ethnicity (non-Hispanic white, non-Hispanic black, Hispanic, other), education (<HS, HS or some college, college graduate or above [College grad]), SNAP, and income (PIR < 1.3 or ≥ 1.3) within Medicare; by race/ethnicity, education, SNAP, and age (35–54, 55–74, ≥75 years) within Medicaid; and by race/ethnicity, education, and SNAP within dual-eligible participants. Income strata are not shown in Medicaid and dual-eligible because adults on those insurance categories are low-income individuals. HS, high school; ICER, incremental cost-effectiveness ratio; PIR, poverty–income ratio; QALY, quality-adjusted life year; SNAP, Supplemental Nutrition Assistant Program; SNAP-I, SNAP-ineligible individuals; SNAP-NP, SNAP-eligible nonparticipants; SNAP-P, SNAP participants.

Findings were generally robust according to beneficiary characteristics within each insurance group, including by age, race, education, income, and SNAP status (Fig 4). From a healthcare perspective, lifetime ICERs for all subgroups were well below $50,000/QALY. For the healthy food incentive, within Medicare ICERs ranged from $9,303/QALY among college graduates to $14,116/QALY among individuals of lower than high-school education; within Medicaid, ICERs ranged from $7,176/QALY among whites to $23,802/QALY in college graduates; and within dual-eligible, ICERs ranged from $3,582/QALY in SNAP-ineligible individuals to $16,365/QALY in SNAP-eligible nonparticipants. Corresponding ICERs were modestly lower from a societal perspective and modestly higher for the F&V incentive (S1 Appendix Table O). Within Medicare, lifetime ICERs for both incentive programs were also highly cost-effective in individuals aged <65 years and ≥65 years (e.g., $8,119/QALY and $19,991/QALY, respectively, for the healthy food incentive) (S1 Appendix Tables P-Q).

Excluding seafood and plant oils from the healthy food incentive, estimated lifetime health gains included 2.83 million CVD events, 0.53 million CVD deaths, and 0.13 million diabetes cases averted and 7.15 million QALYs gained (S1 Appendix Table R). Policy costs to implement the healthy food incentive would be $162.3 billion, with net costs of $80.1 billion from a healthcare perspective and $56.7 billion from a societal perspective. From a healthcare perspective, overall population ICER would be $11,201/QALY, and by insurance group ICER was modestly higher for Medicare ($11,388/QALY) versus Medicaid ($9,239/QALY) and dual-eligible ($7,206/QALY). Corresponding lifetime ICERs from a societal perspective would be $7,920/QALY, $8,449/QALY, $4,181/QALY, and $2,584/QALY, respectively.

In one-way sensitivity analyses testing lower (20%) and higher (50%) food subsidy levels, projected changes in CVD events and deaths, diabetes cases, QALY gains, healthcare savings, and net costs accordingly decreased or increased, but all subsidy levels were highly cost-effective (e.g., $9,870/QALY and $19,184/QALY for the 20% and 50% subsidy levels for the healthy food incentive, respectively) (S1 Appendix Tables S-U, Fig D).

## Discussion

Based on a validated microsimulation model incorporating nationally representative data, a healthcare program incentivizing specific healthful foods would generate significant health gains among adults on Medicare and Medicaid. Over a lifetime, a 30% F&V incentive was estimated to prevent 1.93 million CVD events and gain 4.64 million QALYs, whereas a broader 30% healthy food incentive was estimated to prevent 3.28 million CVD events and 0.12 million diabetes cases and gain 8.40 million QALYs. Both scenarios were cost-effective at 5 years and highly cost-effective at 10 and 20 years and over a lifetime. Incorporating additional savings from productivity gains and informal healthcare costs, these programs would be even more cost-effective with lifetime overall population ICERs of $14,576/QALY and $9,497/QALY, respectively. To our knowledge, this is the first study to assess the health impacts, costs, and cost-effectiveness of incentivizing diet through US health insurance programs.

Our findings were robust across different beneficiary groups, including for Medicare, Medicaid, and dual-eligible beneficiaries. At 5 and 10 years, both incentive programs were modestly more cost-effective within Medicare and dual-eligible compared with Medicaid populations. For example, 5-year ICERs for the healthy food incentive from a societal perspective were about $72,000–$75,000/QALY for Medicare and dual-eligible versus $126,000/QALY for Medicaid. This is consistent with older ages of Medicare and dual-eligible enrollees (mostly aged 65+ years) versus Medicaid adult enrollees (mean 54 years), resulting in larger absolute reductions in cardiometabolic events and subsequent healthcare savings over a shorter timeframe. In contrast, at 20 years and over a lifetime, the incentive programs were modestly more cost-effective in Medicaid than Medicare, with lifetime ICERs of about $6,000/QALY for Medicaid versus $10,000/QALY for Medicare. This suggests that healthcare savings and productivity gains would be larger among younger adults over a longer timeframe. Nonetheless, these incentives were cost-effective at 5 years for all the insurance groups and highly cost-effective at 20 years and beyond. Similarly, both incentive programs were highly cost-effective across subgroups within each insurance group at 20 years and beyond, including by age, race/ethnicity, education, income, and SNAP status. For Medicare beneficiaries under 65 years of age, which is about 16% of total Medicare enrollees [48] who may be particularly vulnerable because of disability or other underlying factors [49], both programs remained highly cost-effective.

Our findings support the implementation of pilot and demonstration programs to test this novel and promising approach in Medicare and Medicaid. For example, the Center for Medicare and Medicaid Innovation [50] could develop and test a pilot program to examine the feasibility of incentivizing healthful foods through Medicare and Medicaid. In recent years, two health-improving incentive programs have been proposed for Medicare and Medicaid beneficiaries [6,7], both targeting traditional cardiometabolic risk factors such as body weight, diabetes control, cholesterol, smoking, and blood pressure, with one of these now implemented in for Medicaid beneficiaries 10 states [6]. Given that nearly half of all cardiometabolic deaths are linked to poor diet [11], our findings suggest that incorporating incentives for healthier eating into such programs could further improve the health of Medicare and Medicaid beneficiaries. The 2018 Farm Bill authorized up to $25 million for a new Produce Prescription Program to test and evaluate interventions in healthcare using financial and other incentives to encourage F&V consumption among patients. Our findings have direct implications for the design and evaluation of interventions funded under this new Produce Prescription Program.

New public–private partnerships could also support economic incentives for healthy food choices: for example, Wholesome Wave’s Fruit and Vegetable Prescription Program (FVRx) operates through collaborations between Wholesome Wave, healthcare providers, and local farm-to-retail businesses to provide $1 coupons for physician-written F&V prescriptions [12]. An evaluation of this program found significant increases in F&V consumption among participating patients, as well as reductions in BMI [51]. This year, the state of California launched a $6 million pilot intervention in their Medicaid (Medical) program to cover medically tailored meals [52], indicating interest and willingness by government insurance programs to incorporate and evaluate novel nutrition programs.

Average estimated subsidy costs for the F&V incentive and healthy food incentive programs were about $110 and $185 per individual per year, respectively. From a healthcare perspective, such approaches promoting healthy eating were estimated to be as or more cost-effective than many currently covered medical interventions, such as drug treatment for hypertension ($20,000/QALY) [53], use of statins for primary prevention ($37,000/QALY) [42], or addition of proprotein convertase subtilisin/kexin type 9 (PCSK9) inhibitor to statins in patients with CVD ($414,000/QALY) [54] or heterozygous familial hypercholesterolemia ($503,000/QALY) [54]. Given that economic incentives through Medicare and Medicare are being considered to promote health, our results highlight the need to prioritize diet as a key component to improve outcomes within health insurance programs. The F&V incentive, healthy food incentive, and modified healthy food incentive (excluding seafood and plant oils) programs were each cost-effective, providing some flexibility in choosing between incentive strategies based on local context. When we evaluated the impact of differential food subsidy levels, all subsidy levels were cost-effective at 5 years and highly cost-effective after 20 years. A higher subsidy level would prevent greater numbers of CVD and diabetes cases and gain more QALYs, suggesting consideration of higher subsidy levels to maximize health benefits.

Our study has several strengths. We used a validated microsimulation model and nationally representative data, which increases confidence in validity of our estimates. Because the estimated changes in food consumption were based on proportional changes in individuals’ current baseline intakes, this implicitly accounts—at least partly—for existing individual characteristics and potential limitations for purchasing healthier foods from supermarkets and grocery stores, for example, based on SNAP participation/eligibility or variations in physical limitations, access, transport, etc. Etiologic effects of dietary changes were estimated from meta-analyses with confirmatory validity analyses, including from randomized trials [11]. We only evaluated proportions of foods purchased at stores that accept EBT cards (e.g., excluding food from restaurants or cafeterias), providing a more appropriate and conservative estimate of potential impact. We assessed both shorter- and longer-term health effects, costs, and cost-effectiveness, providing a range of results across different potential time periods of interest, as well from both healthcare and societal perspectives.

Potential limitations should be considered. Our model cannot prove the health and cost effects of these food incentive programs through Medicare and Medicaid. Rather, the estimates provide evidence that can be considered and incorporated into the design and evaluation of incentive programs at federal (for Medicare and Medicaid) and state (for Medicaid) levels. Etiologic effects of each dietary factor were derived from meta-analyses of prospective observational studies [9,11], which may be overestimated because of residual confounding or underestimated because of measurement error and regression dilution bias. Yet, magnitudes of these estimated etiologic effects are supported by validity analyses of predicted effects based on evidence from randomized controlled feeding studies of CVD risk factors as well as a large randomized clinical trial (i.e., the PREDIMED trial) [11]. We did not model improved health outcomes or cost savings from reductions in other diseases that may be influenced by healthier eating (e.g., cancer, other obesity-mediated conditions), which may underestimate observed benefits. Our investigation did not evaluate political or legal feasibility, which could be considered in future research.

In conclusion, our findings suggest that incentive programs for healthier eating among adults on Medicare and Medicaid could generate substantial health gains and be cost-effective overall and by insurance group. These results have implications for ongoing policy discussions at the federal and state level, as well as for the new federal Produce Prescription Program, for novel approaches to improve health outcomes and reduce healthcare costs through existing health insurance programs.

## Supporting information

S1 Appendix Analysis plan, model description and validation, key modeling assumptions, model inputs, and results of sensitivity analyses. (DOCX)

## Abbreviations

ACC/AHA: American College of Cardiology and American Heart Association
ADA: American Diabetes Association
CHD: coronary heart disease
CMS: Centers for Medicare and Medicaid Service
CVA: cerebrovascular accident
CVD: cardiovascular disease
EBT: electronic benefits transfer
F&V: fruits and vegetables
Food-PRICE: Food Policy Review and Intervention Cost-Effectiveness
FVRx: Fruit and Vegetable Prescription Program
HDL: high-density lipoprotein
HIP: Healthy Incentives Pilot
ICER: incremental cost-effectiveness ratio
LDL: low-density lipoprotein
NHANES: National Health and Nutrition Examination Survey
oz: ounce
PCSK9: proprotein convertase subtilisin/kexin type 9
PIR: poverty–income ratio
PUFA: polyunsaturated fatty acids
QALY: quality-adjusted life year
SNAP: Supplemental Nutrition Assistant Program
USDA: United States Department of Agriculture
