# Supplementary material for: Cost-effectiveness of financial incentives for improving diet and health through Medicare and Medicaid: A microsimulation study
Source: PLoS Med. 2019 Mar 19;16(3):e1002761. doi: 10.1371/journal.pmed.1002761 (PMC6424388; doi:10.1371/journal.pmed.1002761)
Supplement: S1 Appendix — (DOCX) [file pmed.1002761.s001.docx]

Supplementary Appendix

This appendix has been provided by the authors to give readers additional information about their work.

Supplement to: Lee Y, Mozaffarian D, Sy S et al. Cost-effectiveness of Financial Incentives for Improving Diet and Health through Medicare and Medicaid: A Microsimulation Study

CONTENTS

[**Text A.** Analysis plan for the study of cost-effectiveness of financial incentives for improving diet and health through Medicare and Medicaid 4](#_Toc2096759)

[**Text B.** Assessment of validity and bias in diet-disease etiologic effects 6](#_Toc2096760)

[**Text C.** CVD PREDICT microsimulation model 9](#_Toc2096761)

[***Model Description*** 9](#_Toc2096762)

[***Model Validation*** 10](#_Toc2096763)

[**Text D**. Policy costs for a healthful food incentive program Medicare and Medicaid 12](#_Toc2096764)

[**Table A**. Key modeling assumptions 14](#_Toc2096765)

[**Table B**. Food categories for financial incentives through Medicare and Medicaid 15](#_Toc2096766)

[**Table C**. Intervention effect sizes of financial incentives through Medicare and Medicaid 16](#_Toc2096767)

[**Table D**. Estimated age-specific etiologic effects of dietary components on cardiometabolic outcomes 17](#_Toc2096768)

[**Table E**. Comparison of Relative Risks for CHD Observed in Prospective Cohort Studies of Dietary Patterns and Estimated Relative Risks for Individual Dietary Factors. 18](#_Toc2096769)

[**Table F**. Comparison of Relative Risks for CHD Calculated Based on Changes in Systolic Blood Pressure and LDL-cholesterol in Randomized Controlled Feeding Trials of Dietary Patterns vs. Estimated Relative Risks for Individual Dietary Factors 20](#_Toc2096770)

[**Table G.** Comparison of Relative Risks for CHD Observed in a Large Randomized Clinical Trial of Dietary Patterns vs. Estimated Relative Risks for Individual Dietary Factor. 22](#_Toc2096771)

[**Table H**. Estimated intervention costs to implement a financial incentive program to promote intake of healthful foods through Medicare and Medicaid. 23](#_Toc2096772)

[**Table I**. Estimated formal healthcare, informal healthcare, and productivity costs 24](#_Toc2096773)

[**Table J.** Model Inputs for Probabilistic Sensitivity Analysis 26](#_Toc2096774)

[**Table K**. Baseline demographic and health characteristics, and dietary intakes of U.S. adults on Medicare and Medicaid aged 35-80 years. 28](#_Toc2096775)

[**Table L**. Baseline consumption levels and estimated changes in consumption per person at 1 year for individuals on Medicare and/or Medicaid for the two policy scenarios: results of probabilistic sensitivity analyses 30](#_Toc2096776)

[**Table M**. Lifetime health gains, costs, and cost-effectiveness of 30% F&V incentive and healthy food incentive programs through Medicare and Medicaid from a societal perspective 31](#_Toc2096777)

[**Table N.** Results of probabilistic sensitivity analyses at 5 years 33](#_Toc2096778)

[**Table O**. Lifetime estimated health gains, costs, and cost-effectiveness of the 30% F&V and health food incentive programs, by race/ethnicity, education, Supplemental Nutrition Assistance Program, income and age 35](#_Toc2096779)

[**Table P**. Lifetime health gains, costs, and cost-effectiveness of 30% F&V incentive and healthy food incentive programs among Medicare beneficiaries by age 39](#_Toc2096780)

[**Table Q**. Lifetime health gains per million persons of 30% F&V incentive and healthy food incentive programs among Medicare beneficiaries by age group 40](#_Toc2096781)

[**Table R**. Sensitivity analyses of lifetime health gains, costs, and cost-effectiveness of a 30% healthy food incentive program excluding seafood and plant oils through Medicare and Medicaid 41](#_Toc2096782)

[**Table S**. Lifetime health gains, costs, and cost-effectiveness of 20% F&V incentive and healthy food incentive programs through Medicare and Medicaid 43](#_Toc2096783)

[**Table T.** Lifetime health gains, costs, and cost-effectiveness of 50% F&V incentive and healthy food incentive programs through Medicare and Medicaid 45](#_Toc2096784)

[**Table U**. Lifetime health gains per million persons of 20, 30, and 50% F&V incentive and healthy food incentive programs through Medicare and Medicaid 47](#_Toc2096785)

[**Fig A.** Logic pathway linking the healthy food incentive program through Medicare and Medicaid to changes in cardiometabolic health outcomes, QALYs, and health-related costs. 49](#_Toc2096786)

[**Fig B**. Estimated reductions in total CVD events averted (Panel A), diabetes cases averted (Panel B), QALYs (Panel C), healthcare savings (Panel D), net costs (Panel E), and ICERs (Panel F) of the 30% F&V incentive program through Medicare and Medicaid by insurance type over 5, 10, 20 years and lifetime from a healthcare perspective 50](#_Toc2096787)

[**Fig C**. Estimated health-related savings, net costs, and incremental cost-effectiveness ratio of the 30% F&V incentive and healthy food incentive programs through Medicare and Medicaid by insurance over 5, 10, 20 years and lifetime from a societal perspective 51](#_Toc2096788)

[**Fig D.** Estimated reductions in total CVD events averted (Panel A), diabetes cases averted (Panel B), quality-adjusted life years (QALYs) (Panel C), healthcare savings (Panel D), subsidy costs (Panel E), net costs (Panel F) and incremental cost-effectiveness ratio (Panel G) of the 20, 30, and 50% healthy food incentive program through Medicare and Medicaid over 5, 10, 20 years and a lifetime 53](#_Toc2096789)

[**References** 54](#_Toc2096790)

# **Text A.** Analysis plan for the study of cost-effectiveness of financial incentives for improving diet and health through Medicare and Medicaid

**Policy scenarios**

We will model two distinct interventions within the Medicare/Medicaid program, compared with a base-case of no new intervention: 1) 30% subsidy on fruits and vegetables (F&V incentive), and 2) 30% subsidy on broader healthful foods (healthy food incentive) including F&V, whole grains, nuts/seeds, seafood, and plant oils. A secondary analysis will be performed excluding incentives for seafood and plant oils, the two most expensive food categories, from the healthy food incentive.

**Target population**

Our population will be based on US adults aged 35-80 years at baseline across three cycles of NHANES (2009-2014) enrolled in Medicare and/or Medicaid, defined by reporting Medicare and/or Medicaid insurance coverage in the health insurance questionnaire. In addition, data on the CVD risk factors necessary to run the CVD PREDICT model will be obtained: sex, age, systolic blood pressure, total cholesterol, HDL cholesterol, and smoking and diabetes statuses.

**Time horizons**

We will estimate the health and economic impacts of the 1) F&V incentive and 2) healthy food incentive programs over 5 (2018-2022), 10 (2018-2027), 20 (2018-2037) years and a lifetime to investigate the short-, mid-, long-term impacts of the interventions.

**Perspectives**

In accordance with recommendations from the Second Panel on Cost-Effectiveness in Health and Medicine,[1] analyses will be conducted from two perspectives: (1) healthcare perspective, incorporating policy costs and formal healthcare costs; and (2) societal perspective, further incorporating informal healthcare and productivity costs.

**Microsimulation model and outcome measures**

CVD-PREDICT is a validated micro-simulation model coded in C++, which has been used to simulate and quantify effects of policies on coronary heart disease, stroke, and diabetes.[2-4] The model will be populated with simulated individuals on Medicare and/or Medicaid including their risk factors such as age, sex, systolic blood pressure, total cholesterol, HDL cholesterol, smoking, diabetes and current dietary habits from NHANES 2009-2014. At any given time point, a simulated individual could be one health state, with the probability of experiencing subsequent events based on each person’s cardiometabolic risk factors and dietary habits.

Model outputs include CVD events, CVD deaths, diabetes cases, quality-adjusted life years, event-associated health-related costs, and cost-effectiveness at 5, 10, 20 years and cohort lifetime.

**Stratified analyses**

To investigate consistency of health and economic impacts of each program across subgroups within each insurance group, analyses will be further stratified by race/ethnicity (non-Hispanic white, non-Hispanic black, Hispanic, other), education (<high school, high school or some college, college graduate or above), Supplemental Nutrition Assistance Program (SNAP) (SNAP participants, SNAP eligible, non-participants, SNAP ineligible individuals), and income (PIR<1.3 or ≥1.3) within Medicare; by race/ethnicity, education, SNAP, and age (35-54, 55-74, ≥75 years) within Medicaid; and by race/ethnicity, education, and SNAP within dual-eligible participants.

**Probability sensitivity analyses**

We will assess the potential impact of uncertainty in key model parameters with given input probability distributions. The key parameters include uncertainty in policy effect sizes, diet-disease relative risks, CVD risks, food unit costs, formal and informal healthcare costs, and utility weights. Once thousand simulations will be run drawing from the uncertainty distributions of each of these inputs at 5 years and lifetime, with 95% uncertainty intervals based on the 2.5th and 97.5th percentiles of the 1,000 simulations.

**Text B.** Assessment of validity and bias in diet-disease etiologic effects **[**adapted with permission from Micha R, Shulkin ML, Penalvo JL, et al. Etiologic effects and optimal intakes of foods and nutrients for risk of cardiovascular diseases and diabetes: Systematic reviews and meta-analyses from the Nutrition and Chronic Diseases Expert Group (NutriCoDE). *PLoS One*. 2017;12(4):e0175149.]

While nearly all identified observational studies in the utilized meta-analyses utilized multivariable adjustment for major demographics, CVD risk factors and, in many cases, other dietary factors,[5] we recognized that clustering of dietary patterns could still cause unmeasured confounding, e.g., from clustering of healthful factors such as fruits, vegetables, and whole grains. Thus, even with multivariable adjustment, our final calculated etiologic effects from studies of an individual dietary component might overestimate its effects, as compared with the true effect when the dietary component is consumed as part of an overall diet pattern.

To assess potential bias from dietary pattern effects, we performed 3 validity analyses including based on: (a) prospective long-term observational studies evaluating overall dietary patterns and clinical cardiovascular events; (b) randomized controlled feeding trials evaluating overall dietary patterns and cardiovascular risk factors (LDL-cholesterol, SBP); and (c) a large RCT evaluating overall dietary patterns and clinical cardiovascular events. For each, we compared the observed effect from the dietary pattern study to the estimated RR calculated by jointly considering the individual etiologic effects (RRs) for each dietary component in that pattern.

For prospective cohorts evaluating overall diet patterns and CVD events,[6-10] the observed multivariable-adjusted RR in each category (e.g., quintile) of the dietary pattern was compared to the estimated effect calculated by combining the reported differences in each individual dietary component (e.g., fruits, nuts) across each category of the diet pattern with our estimated individual etiologic effect (RR) for that dietary component, assuming a multiplicative relation between RRs for individual components. We focused on foods and excluded overlapping components (e.g., we included whole grains, fruits, and vegetables; and excluded dietary fiber); we also assumed no benefits from differences in other dietary factors (e.g., coffee) in the pattern for which we had not determined a causal etiologic effect.

For randomized controlled feeding trials of dietary patterns and CVD risk factors, we performed inverse-variance-weighted meta-regression across all of the treatment arms of three large, well-established dietary pattern trials [11-13] to estimate the independent effects of five individual dietary components, when consumed as part of an overall dietary pattern, on SBP and LDL-cholesterol. We evaluated achieved changes in fruits, vegetables, nuts, whole grains, and fish simultaneously as independent variables, with changes in SBP or LDL-C as the dependent variable. For each dietary component, we then calculated how the identified change in SBP and LDL-C from the meta-regression would alter cardiovascular risk, based on the established relationship between SBP and LDL-C and clinical events [14-18] assuming independent, multiplicative effects of SBP and LDL-C. These effects, calculated based only on how each dietary component altered SBP and LDL-C in randomized controlled feeding trials of diet patterns, were then compared to our estimated etiologic effect on cardiovascular events for that dietary component. We recognized that the calculated effects based on the feeding trial results might be conservative, as they presume that the summed CVD benefits of these dietary factors are attributable only to effects on SBP and LDL-C, when in reality other pathways of benefit likely exist.

Lastly, we compared the observed vs. estimated risk using findings from the PREDIMED trial, a large RCT evaluating the effects of two overall dietary patterns on CVD incidence.[19] The estimated risk reductions were calculated by combining the observed differences in individual dietary components achieved in the trial with our estimated quantitative effects for each dietary component, assuming multiplicative effects of each individual component.

Each of these validity analyses demonstrated that the estimated etiologic effects for individual dietary components were very similar to what would be expected based on these other lines of evidence.[5] (S1 Appendix Tables E-G).

The related question of dietary complements and substitutes was also considered and discussed at length during our study design. In brief, because the diet-disease etiologic effects (relative risks) for the changes in the targeted dietary factors are based on long-term prospective cohort studies, the estimates already incorporate all of the average substitutes and complements across the population. In other words, a cohort study comparing risk associated with higher vs. lower fruit intake already implicitly incorporates the varying dietary substitutes and complements associated with the observed difference in risk, in that people with lower fruit intake may have more other dietary components, for example. If our intervention strategies aimed to encourage or discourage specific complements and substitutes, the efficacy could theoretically be augmented above the currently calculated average effect.

# **Text C.** CVD PREDICT microsimulation model

## ***Model Description***

The CVD PREDICT model simulates aging and health transitions of individual adults using a variety CVD-related epidemiological data, including adapted Framingham-based risk scores for ischemic heart disease and stroke, calibrated and validated to observed risk in the US adult population. The model uses a micro-simulation approach to more accurately model the natural history of CVD. Specifically, the ability to store and modify specific CVD risk factors on an individual basis and update individual-level CVD disease history are great strengths of the model. Full details of the CVD PREDICT Model and validation result have been published elsewhere.[20]

Briefly, the CVD PREDICT model is populated with a database of individual adults age 35+ years with accompanying risk factor data. The CVD risk factors used to predict risk of disease transitions include age, sex, systolic blood pressure, total cholesterol, HDL cholesterol, smoking status, and diabetes status. The model also considers an individual’s prior history of having a CVD event and populates these individuals in their respective CVD health states at the start of each 1-year model run. To achieve national representativeness, adult participants from the National Health and Nutrition Examination Surveys (NHANES) are sampled with replacement using NHANES sample weights to create a representative population of 1,000,000 individuals. Risk factors and dietary distributions are therefore directly embedded in the model population based on NHANES reports all of the variables at baseline. Descriptive statistics for the model population (S1 Appendix Table K) were generated using the statistical software packages STATA 14.

In each yearly time frame, three main updates occur: updating of the risk factors (e.g. an increase in systolic blood pressure), potential transitions into a different CVD health state, and preventive interventions (e.g. screening, medication, lifestyle modifications, or as in this case, food and nutrition policy intervention). These annual simulations are repeated for the duration of the time period of interest (e.g., 5 years, 10 years, 20 years, lifetime), with a closed population design.

For each intervention scenario as well as no new intervention (natural history), the model generates the predicted changes in probability of each health outcome at the individual level, both overall and stratified by insurance group (Medicare, Medicaid, dual-eligible). To investigate consistency of the health and economic impacts of each program across subgroups within each insurance group, analyses were further stratified by age (35-64, ≥65 years), race/ethnicity (non-Hispanic white, non-Hispanic black, Hispanic, other), and education (< high school, high school graduate/some college, college graduate or above) and Supplemental Nutrition Assistance Program (SNAP) (SNAP participants, SNAP eligible, non-participants, SNAP ineligible individuals), and income (PIR<1.3 or ≥1.3) within Medicare; by race/ethnicity, education, SNAP, and age (35-54, 55-74, ≥75 years) within Medicaid; and by race/ethnicity, education, and SNAP within dual-eligible participants.

Health states, events, and costs are then determined based on each yearly population simulation. The CVD PREDICT model tracks each cardiometabolic event (CVD, diabetes) for every individual run through the model, including the number of deaths arising from each type of event, the average life expectancy of those with and without CVD, the number of individuals who have ever had an event, and the yearly prevalence of every disease state. Additionally, as costs and health state utilities are accrued by each individual in every (yearly) model cycle, the model also produces event-associated and overall healthcare costs, and quality-adjusted life years (QALYs) which capture both number and quality of years survived by each individual. Utility values ranging between 0 (death) and 1 (perfect health) were assigned to each health state (disease free, cardiac arrest, myocardial infarction, angina, stroke, death) based on EuroQOL 5 Dimensions (EQ-5D) questionnaire results from the Medical Expenditure Panel Survey (MEPS).[21] Population totals and averages are calculated and stored once the appropriate number of individuals complete simulation over the relevant time period. We provide additional data incorporating productivity gains and losses using US data to capture a societal perspective

## ***Model Validation***

We have previously calibrated and validated our micro-simulation model using recent NHANES data with a population from 1999-2011.[20] Model-based (simulated) results were compared to observed all-cause and CVD specific mortality for the same starting population using survival curves and receiver operating characteristic curves (Available from <http://journals.sagepub.com/doi/abs/10.1177/0272989X17706081?journalCode=mdma>).  Five-year and 10-year CVD and all-cause mortality from the CVD PREDICT model fell within the 95% CIs of the observed data. For example, observed 10-year all-cause mortality in NHANES versus the simulation model was 11.2% (95% CI, 10.3% to 12.2%) v. 10.9%; corresponding results for CVD mortality were 2.2% (1.8% to 2.7%) vs. 2.6%. CVD PREDICT model-based all-cause mortality projections at 20 and 30 years were 27.7% and 47.8%; the corresponding results for the life table extrapolation of the same starting population were 28.1% and 48.9%, respectively. Life expectancy was 82.5 years and 81.7 years for the CVD PREDICT model-based and life table-based projections, respectively. Areas under the ROC curves for model-predicted 10-year all-cause and CVD mortality risks were 0.83 (0.81 to 0.85) and 0.84 (0.81 to 0.88), respectively; corresponding results for 5-year risks were 0.80 (0.77 to 0.83) and 0.81 (0.75 to 0.87), respectively.

Because the predictive risk functions that determine the model risk for CVD (Framingham risk score, AHA/ACC pooled cohort ASCVD risk score) were developed primarily for adults age 35 to 79 years, the model was not extended to younger ages, which could reduce validity. Notably, because very few events occur before the age of 35 years (0.6% among those 20-39 compared with 5-30% in older age groups), excluding very young adults would have little influence on findings.

**Text D**. Policy costs for a healthful food incentive program Medicare and Medicaid.

Policy costs included the administrative costs of the program implementation, and the subsidy costs for incentivized foods. To estimate the administrative costs, we considered sources from SNAP and Medicaid given similarities to the proposed intervention design and population of interest. The 20% of the total subsidy costs was assumed to be the administrative costs in the first year ($1.8 billion), conservatively based on the administrative costs (i.e., the percentage of total benefits) for the SNAP program in the first year when the EBT system was introduced.[22] This amount would include many other existing administrative costs of SNAP beyond the set-up of the EBT system. No data are available on the incremental administrative costs of the EBT system in SNAP after the first year.

The Healthy Incentives Program (HIP) trial, which set up a new food subsidy system within SNAP, found that most of the implementation costs were one-time costs such as for system design, development and testing, retailer recruiting and relations, training, and general administration.[23] In the HIP report, they further estimated the overall administrative costs for implementing the program nationwide: the estimated administrative implementation costs in the first year ($96 million) would be 6.2% of total subsidy costs in the first full year of implementation with all retailers participating ($1.6 billion). While administrative implementation costs would likely decrease meaningfully after the first year, we conservatively used 5% for administrative costs in year 2 and beyond (~$450 million per year). We did not formally include alternative cost estimates in sensitivity analyses, because the implementation costs were comparatively small, while the healthcare costs and subsidy costs under the study were more influential for our results.

The costs for food incentives were calculated using the following equation:

$$Subsidy costs =Postintervention intake of healthful foods \times Unit food cost\times Subsidy rate$$

$$Postintervention intake= Changes in intake (baseline intake\times price elasticity*)+Baseline intake$$

*Price elasticity is estimated based on the level of subsidy.

The unit food costs were derived from data from USDA Economic Research Service Quarterly Food-at-Home Price Database.[24] It is worth noting that the subsidy costs would not be increased proportionally to the subsidy rate. By the nature of the intervention, the post-intervention intake of healthful foods already accounted for the subsidy level (i.e., price elasticity), resulting in a non-linear increase in subsidy costs (please see S1 Appendix Fig D, Panel E for the non-linear increase in subsidy costs at a higher subsidy rate).

# **Table A**. Key modeling assumptions

| **Model input** | **Assumption** |
| --- | --- |
| Baseline characteristics | - We assumed NHANES to be representative of the U.S. population. |
| Policy effects | - The time lag that occured from the policy implementation to the dietary changes was assumed to be less than year, and the intervention effect sustained throughout the simulated period. - Policy effects were assumed to differ by income status, but not other population characteristics (e.g., age, sex). |
| Diet-disease etiologic effects (relative risks) | - Diet-disease etiologic effects do not vary by sex. - Diet-disease etiologic effects vary by age. |
| Probability of individual’s health transition | - The total incidence of CHD was assumed to be equal to the sum of the incidence of MI and angina. |
| Utilities | - The utilities of myocardial infarction (MI)-coronary artery bypass graft (CABG) and angina-CABG were assumed to have an equal utility with MI and angina, respectively. - Due to lack of utility data specifically for acute disease states (i.e., within one year of the event), it was assumed that an additional (acute) disutility for the first year of experiencing the CVD event quantified by the regression coefficient for condition-specific disutility from Sullivan et al.[25]. For acute cardiac arrest, we assumed the same additional disutility as MI (-0.0409). |
| Policy costs | - Administrative costs were assumed to be higher in the first year of implementation due to one-time startup costs and lower in subsequent years. |
| Health-related costs | - The disease-free state was assumed to incur no CVD-related costs. |

**Table B**. Food categories for financial incentives through Medicare and Medicaid.^a^

| **Food Category** | **Included Foods** | **Excluded Foods** |
| --- | --- | --- |
| Fruits | - Fresh, frozen, cooked, canned, dried fruits | - Fruit flavored drinks - Salted or pickled fruit - Fruit juice |
| Vegetables | - Fresh, frozen, cooked, canned, dried vegetables - Beans, legumes - Tomato sauce - Starchy vegetables except white potatoes | - Ketchup, barbeque sauce, steak sauce - Salted or pickled vegetables (i.e., olives, pickles) - White potatoes - Vegetable juice |
| Whole grains | - All products containing the entire grain kernel (bran, germ, endosperm), such as amaranth, barley (not pearled), brown rice, buckwheat, bulgur, millets, oats, quinoa, dark rye, triticale, whole-grain wheat flour, whole-grain cracked wheat, wild rice, and grain-based products made with 100% whole grains or their flours | - Corn products (i.e. corn flour, corn meal, and popcorn) |
| Nuts/seeds | - Nuts, peanuts, and seeds, including nut, peanut, and seed butters | - Coconut |
| Seafood | - Fish and shellfish | - Battered fish, fish sticks |
| Plant oils | - All plant based oils including vegetable oil, almond oil, coconut oil, corn oil, canola oil, peanut oil, olive oil, rapeseed oil, soybean oil, sesame oil, safflower oil, walnut oil, cottonseed oil, flaxseed oil, sunflower oil, and wheat germ oil | - Palm oil |

^a^ Derived using data from NHANES 2009-2014,[26] based on two 24-hour dietary recalls per person.

**Table C**. Intervention effect sizes of financial incentives through Medicare and Medicaid.^a^

|  | Price elasticity for a 30% price change, % | % Food purchased from stores^b^ | | | | Overall diet policy effect^c^, % | | | |
| --- | --- | --- | --- | --- | --- | --- | --- | --- | --- |
|  |  | Overall^d^ | Medicare^e^ | Medicaid^f^ | Dual Eligible^g^ | Overall^d^ | Medicare^e^ | Medicaid^f^ | Dual Eligible^g^ |
| **Poverty-income ratio < 1.3** | | | | | | | | | |
| Fruits, g/d | 40.5 | 87 | 86 | 88 | 86 | 35.2 | 34.8 | 35.6 | 34.8 |
| Vegetables, g/d | 40.5 | 71 | 69 | 74 | 73 | 28.7 | 27.9 | 30.0 | 29.6 |
| Whole grains, g/d | 40.5 | 94 | 93 | 94 | 91 | 38.1 | 37.6 | 38.1 | 36.8 |
| Nuts/seeds, g/d | 40.5 | 94 | 93 | 95 | 95 | 38.1 | 37.6 | 38.5 | 38.5 |
| Seafood, g/d | 40.5 | 74 | 70 | 80 | 80 | 30.0 | 28.3 | 32.4 | 32.4 |
| Plant oils, g/d | 40.5 | 72 | 71 | 74 | 77 | 29.1 | 28.7 | 30.0 | 31.2 |
| **Poverty-income ratio ≥ 1.3** | | | | | | | | | |
| Fruits, g/d | 34.3 | 90 | 90 | 86 | 88 | 30.9 | 30.9 | 29.5 | 30.2 |
| Vegetables, g/d | 34.3 | 69 | 69 | 75 | 76 | 23.7 | 23.7 | 25.7 | 26.1 |
| Whole grains, g/d | 34.3 | 94 | 94 | 94 | 93 | 32.2 | 32.2 | 32.2 | 31.9 |
| Nuts/seeds, g/d | 34.3 | 92 | 92 | 97 | 100 | 31.6 | 31.6 | 33.3 | 34.3 |
| Seafood, g/d | 34.3 | 60 | 59 | 71 | 62 | 20.6 | 20.2 | 24.4 | 21.3 |
| Plant oils, g/d | 34.3 | 71 | 70 | 74 | 78 | 24.4 | 24.0 | 25.4 | 26.8 |

^a^ Policy elasticity for 30% price change was calculated as three-fold of the effect size (12.4% increase consumption per 10% price reduction) from a meta-analysis of interventional and prospective observational studies.[27] To account for differential price responsiveness by socio-economic status, we incorporated an overall 18.2% higher price-responsiveness for low income individuals as defined by their income eligibility threshold for government food assistance programs (poverty-income ratio of 1.3), based on a meta-analysis that compared low vs high households within high income countries.[28] This provided elasticity estimates for the low income group (40.5% increase consumption per 30% price reduction) and high income group (34.3% increase consumption per 30% price reduction).

^b^ Based on empirical evidence from U.S. adults on Medicare and Medicaid in NHANES 2009-2014. The percentage of each food category from supermarkets, grocery stores, convenience stores, and other stores was calculated as a proportion of the total consumed (i.e., as compared to other locations such as restaurants, worksites, food pantries, etc.), utilizing NHANES survey and sampling weights.[26]

^c^ Overall diet policy effect = (price elasticity for a 30% price change) $\times$ (% Food purchased from stores).

^d^ Includes Medicare only, Medicaid only, and dual eligible beneficiaries.

^e^ Includes Medicare only and dual eligible beneficiaries.

^f^ Includes Medicaid only and dual eligible beneficiaries.

^g^ Beneficiaries on both Medicare and Medicaid

**Table D**. Estimated age-specific etiologic effects of dietary components on cardiometabolic outcomes.^a^

|  | **Cardiometabolic outcome** | **Unit of Effect** | **Estimated Etiologic Effect (95% CI), by Age** | | | | | |
| --- | --- | --- | --- | --- | --- | --- | --- | --- |
|  |  |  | **25-34y** | **35-44y** | **45-54y** | **55-64y** | **65-74y** | **75+y** |
| Fruits | ↓ CHD | RR per 100 g/d | 0.92 | 0.92 | 0.93 | 0.94 | 0.95 | 0.97 |
|  |  |  | (0.87, 0.97) | (0.87, 0.97) | (0.89, 0.97) | (0.91, 0.98) | (0.92, 0.98) | (0.96, 0.99) |
|  | ↓ Ischemic stroke |  | 0.83 | 0.83 | 0.86 | 0.88 | 0.9 | 0.94 |
|  |  |  | (0.76, 0.90) | (0.77, 0.90) | (0.80, 0.92) | (0.83, 0.93) | (0.86, 0.94) | (0.92, 0.96) |
|  | ↓ Hemorrhagic stroke |  | 0.63 | 0.64 | 0.69 | 0.73 | 0.77 | 0.86 |
|  |  |  | (0.49, 0.81) | (0.5, 0.82) | (0.56, 0.84) | (0.61, 0.87) | (0.67, 0.89) | (0.8, 0.92) |
| Vegetables | ↓ CHD | RR per 100 g/d | 0.93 | 0.93 | 0.94 | 0.95 | 0.96 | 0.98 |
|  |  |  | (0.89, 0.97) | (0.9, 0.97) | (0.91, 0.97) | (0.93, 0.98) | (0.94, 0.98) | (0.97, 0.99) |
|  | ↓ Ischemic stroke |  | 0.76 | 0.77 | 0.8 | 0.83 | 0.86 | 0.92 |
|  |  |  | (0.64, 0.9) | (0.66, 0.9) | (0.7, 0.92) | (0.74, 0.93) | (0.78, 0.94) | (0.87, 0.96) |
|  | ↓ Hemorrhagic stroke |  | 0.76 | 0.77 | 0.8 | 0.83 | 0.86 | 0.92 |
|  |  |  | (0.61, 0.95) | (0.62, 0.95) | (0.67, 0.96) | (0.72, 0.96) | (0.76, 0.97) | (0.86, 0.97) |
| Whole grains | ↓ CHD | RR per 50 g/d | 0.95 | 0.95 | 0.96 | 0.97 | 0.97 | 0.98 |
|  |  |  | (0.91, 0.99) | (0.92, 0.99) | (0.93, 0.99) | (0.94, 0.99) | (0.95, 0.99) | (0.97, 0.99) |
|  | ↓ Total stroke |  | 0.88 | 0.88 | 0.9 | 0.91 | 0.93 | 0.96 |
|  |  |  | (0.80, 0.96) | (0.81, 0.96) | (0.83, 0.97) | (0.86, 0.97) | (0.88, 0.98) | (0.93, 0.98) |
|  | ↓ Diabetes |  | 0.83 | 0.83 | 0.86 | 0.88 | 0.90 | 0.94 |
|  |  |  | (0.76, 0.90) | (0.77, 0.90) | (0.80, 0.92) | (0.83, 0.93) | (0.83, 0.94) | (0.92, 0.96) |
| Nuts/seeds | ↓ CHD | RR per 1-oz serving/wk | 0.89 | 0.89 | 0.91 | 0.92 | 0.93 | 0.96 |
|  |  |  | (0.85, 0.93) | (0.85, 0.93) | (0.87, 0.94) | (0.89, 0.95) | (0.91, 0.96) | (0.95, 0.97) |
|  | ↓ Diabetes |  | 0.95 | 0.95 | 0.96 | 0.97 | 0.97 | 0.98 |
|  |  |  | (0.92, 0.98) | (0.93, 0.98) | (0.94, 0.98) | (0.95, 0.98) | (0.96, 0.99) | (0.98, 0.99) |
| PUFA replacing carbs | ↓ CHD | RR per 5%E | 0.86  (0.79, 0.92) | 0.86  (0.8, 0.93) | 0.88  (0.83, 0.94) | 0.9  (0.86, 0.95) | 0.92  (0.88, 0.96) | 0.95  (0.93, 0.97) |
| Seafood omega-3 fats^b^ | ↓ CHD (fatal) | RR per 100 mg/d | 0.79 | 0.80 | 0.82 | 0.85 | 0.87 | 0.93 |
|  |  |  | (0.70, 0.88) | (0.71, 0.89) | (0.75, 0.90) | (0.79, 0.92) | (0.82, 0.93) | (0.90, 0.96) |

^a^ The detailed methods for reviewing and synthesizing evidence to estimate effect sizes for associations between dietary factors and cardiometabolic endpoints have been reported.[5, 29] We utilized evidence from meta-analyses of prospective cohorts or randomized clinical trials evaluating direct associations of dietary factors with coronary heart disease (CHD), stroke, or type 2 diabetes, by age.

^b^ The available evidence suggests an effect of seafood omega-3 on fatal CHD, with less clear evidence for benefits on nonfatal CHD.[30] Because the risk transitions influenced by diet in the CVD-Predict model are for incidence of a CHD event, with subsequent transitions to death independent of dietary risk factors (see Fig 1), the current analysis will modestly overestimate the benefits of changes in seafood omega-3 consumption.

CHD - coronary heart diseases, RR - relative risk.

# **Table E**. Comparison of Relative Risks for CHD Observed in Prospective Cohort Studies of Dietary Patterns and Estimated Relative Risks for Individual Dietary Factors. ^a^

| **Study** | **Estimate type** | **Q2** | **Q3** | | **Q4** | | **Q5** | **Average of all quintiles** ^b^ | **Mean absolute risk difference, calculated vs. observed** ^c^ |
| --- | --- | --- | --- | --- | --- | --- | --- | --- | --- |
| Health Professionals Study – Prudent Dietary Pattern[6] | Calculated RR in each quintile ^d^ | 0.86 | 0.82 | | 0.74 | | 0.63 | 0.75 | 0.07 |
|  | Calculated RR, adjusted for time ^e^ | 0.88 | 0.83 | | 0.76 | | 0.66 | 0.78 | 0.04 |
|  | Observed RR | 0.90 | 0.83 | | 0.79 | | 0.75 | 0.82 |  |
|  |  |  |  | |  | |  |  |  |
| Health Professionals Study – Western Dietary Pattern[6] | Calculated RR in each quintile ^d^ | 1.12 | 1.16 | | 1.19 | | 1.30 | 1.19 | -0.10 |
|  | Calculated RR, adjusted for time ^e^ | 1.10 | 1.14 | | 1.17 | | 1.26 | 1.17 | -0.12 |
|  | Observed RR | 1.21 | 1.27 | | 1.27 | | 1.43 | 1.29 |  |
|  |  |  |  | |  | |  |  |  |
| Nurses’ Health Study – Prudent Dietary Pattern[7] | Calculated RR in each quintile ^d^ | 0.91 | 0.80 | | 0.75 | | 0.66 | 0.77 | 0.05 |
|  | Calculated RR, adjusted for time ^e^ | 0.92 | 0.82 | | 0.77 | | 0.69 | 0.80 | 0.02 |
|  | Observed RR | 0.95 | 0.83 | | 0.76 | | 0.76 | 0.82 |  |
|  |  |  |  | |  | |  |  |  |
| Nurses’ Health Study – Western Dietary Pattern [7] | Calculated RR in each quintile ^d^ | 1.04 | 1.07 | | 1.15 | | 1.23 | 1.12 | -0.08 |
|  | Calculated RR, adjusted for time ^e^ | 1.03 | 1.06 | | 1.13 | | 1.20 | 1.10 | -0.10 |
|  | Observed RR | 1.01 | 1.10 | | 1.26 | | 1.46 | 1.20 |  |
|  |  |  |  | |  | |  |  |  |
| Nurses’ Health Study – Mediterranean Dietary Pattern[8] | Calculated RR in each quintile ^f^ | 0.93 | 0.82 | | 0.76 | | 0.72 | 0.80 | 0.04 |
|  | Calculated RR, adjusted for time ^e^ | 0.93 | 0.83 | | 0.77 | | 0.73 | 0.81 | 0.03 |
|  | Observed RR | 0.92 | 0.87 | | 0.87 | | 0.71 | 0.84 |  |
|  |  | Men | | Women | | Overall | |  |  |
| EPIC Greece – Mediterranean Dietary Pattern[9] | Calculated RR per 2 unit increase ^g^ | 0.90 | | 0.91 | | 0.90 | |  | -0.12 |
|  | Observed RR | 0.81 | | 0.75 | | 0.78 | |  |  |
|  |  |  | | |  | | |  |  |
| SUN Cohort Spain – Mediterranean Dietary Pattern[10] | Calculated RR per 2 unit increase ^h^ | 0.75 | | | | | |  | -0.01 |
|  | Observed RR | 0.74 | | | | | |  |  |
|  |  | | | | | | |  |  |

^a^ The observed multivariable-adjusted relative risk (RR) in each category or per each unit of the dietary pattern was compared to the predicted effect calculated by combining the reported differences in individual dietary factors (including fruit, vegetables, whole grains, fish, processed meat, *trans* fat, polyunsaturated fat) across each category or per unit of the diet pattern with their Nutrition and Chronic Diseases Expert Group (NutriCoDE)-estimated individual quantitative effects, assuming a multiplicative relation between RRs for individual components. We primarily utilized the RRs for foods and excluded overlapping components (e.g., whole grains and fiber; or meats and saturated fats) in these analyses. The calculated RRs also assumed no benefits from changes in other dietary factors (e.g., coffee) for which we had not determined a causal etiologic effect, which could cause the observed RRs to be greater than the calculated RRs; and also incorporated the NutriCoDE threshold of optimal intake, beyond which no further benefit was assumed.

*^b^* Based on the mean of beta-coefficients (ln RR’s) across quintiles within each study.

*^c^* Based on the mean absolute risk difference of calculated vs. observed RR’s [(1-calculated RR) - (1-observed RR)]. Compared to observed RRs as the reference, positive values represent overestimation of calculated RRs, while negative values represent underestimation of calculated RRs.

*^d^* Dietary factors reported and included were fruits, vegetables, whole grains, fish, processed meat, *trans* fat, and polyunsaturated fat.

*^e^* Accounting for observed declining dietary differences over time in the dietary pattern studies in these specific cohorts.

*^f^* Dietary factors reported and included were fruit, vegetables, whole grains, omega-3s, processed and red meat, and *trans* fat.

*^g^* Dietary factors reported and included were vegetables plus legumes, fruits and nuts, fish, processed meat, and polyunsaturated fat. The dietary comparisons used were for the 75th vs. 25th percentiles.

^h^ Dietary factors reported and included were vegetables plus legumes, fruits, fish, whole grains, nuts, processed meats, and polyunsaturated fat.

Table adapted with permission from Micha R, Shulkin ML, Penalvo JL, et al. Etiologic effects and optimal intakes of foods and nutrients for risk of cardiovascular diseases and diabetes: Systematic reviews and meta-analyses from the Nutrition and Chronic Diseases Expert Group (NutriCoDE). *PLoS One*. 2017;12(4):e0175149.

**Table F**. Comparison of Relative Risks for CHD Calculated Based on Changes in Systolic Blood Pressure and LDL-cholesterol in Randomized Controlled Feeding Trials of Dietary Patterns vs. Estimated Relative Risks for Individual Dietary Factors.

| **Dietary risk factor** | **Change in SBP (mmHg) in dietary feeding RCTs** *^a^* | **Change in LDL-C (mg/dL) in dietary feeding RCTs** *^a^* | **Predicted RR of CHD in dietary feeding RCTs, based on SBP effect** *^b^* | **Predicted RR of CHD in dietary feeding RCTs, based on LDL-C effect** *^b^* | **Multiplicative RR of CHD per serving in dietary feeding RCTs, based on joint SBP and LDL-C effects** | **Estimated RR of CHD from cohort studies**  **(NutriCoDE relative risks, see Table 2)** |
| --- | --- | --- | --- | --- | --- | --- |
| Fruits, per serving/d  (100 g/d) | -0.33 | -1.5 | 0.99 | 0.94 | 0.93 | 0.94  (0.91, 0.98) |
| Vegetables, per serving/d  (100 g/d) | -0.18 | -1.6 | 0.99 | 0.94 | 0.93 | 0.95  (0.92, 0.98) |
| Nuts/seeds, per serving/wk  (28.35 g) | -0.92 | -1 | 0.97 | 0.96 | 0.93 | 0.93  (0.91, 0.96) |
| Whole grains, per serving/d  (50 g/d) | -0.11 | -3.2 | 1.00 | 0.88 | 0.88 | 0.97  (0.94, 0.99) |
| Fish, per serving/d  (100 g/d) | N/A*^c^* | -3.4 | N/A *^c^* | 0.87 | 0.87 | 0.66  (0.50, 0.87) |
| Red meat, per serving/d  (100 g/d) | 3.20 | 1.1 | 1.12 | 1.04 | 1.17 | 1.17  (1.05, 1.30) *^d^* |
| Dietary fiber, per 20 g/d  (20 g/d) *^e^* | -3.00 | -3.9 | 0.89 | 0.86 | 0.77 | 0.76  (0.68, 0.85) |

*^a^* For systolic blood pressure (SBP), trials include OmniHeart (protein diet vs. baseline diet, carbohydrate diet vs. baseline diet, and unsaturated fat diet vs. baseline diet),[13] DASH-sodium (high sodium DASH diet vs. high sodium control diet),[12] and DASH (combination diet vs. control diet, fruit and vegetable diet vs. control diet).[11] For LDL-C, trials include OmniHeart (protein diet vs. baseline diet, carbohydrate diet vs. baseline diet, and unsaturated fat diet vs. baseline diet), DASH-sodium (high sodium DASH diet vs. high sodium control diet, intermediate sodium DASH diet vs. intermediate sodium control diet, low sodium DASH diet vs. low sodium control diet). Results reflect pooled meta-regression models simultaneously accounting for all dietary changes in these dietary patterns trials, i.e. changes in each dietary factor in this Table.

*^b^* Based on the observed association between SBP and incident coronary heart disease (CHD) events and LDL-C and incident CHD events in large pooling projects of prospective cohort studies.[6, 7, 31]

*^c^* Analysis of the impact of including fish in the blood pressure meta-regression model indicated that small changes in fish intake caused improbably large changes in blood pressure. Therefore, fish was not included in the blood pressure meta-regression.

*^d^* Based on prospective cohort studies, we identified evidence for an etiologic relative risk (RR) for CHD for processed meat, but not unprocessed red meat. Because these feeding studies evaluated only total meat consumption, the corresponding RR for cohort studies represents the estimated RR for total meat consumption, based on approximately 25% of total meat consumption being processed meat, a 100 g serving size, and assuming no significant etiologic effect of unprocessed red meat.

*^e^* Due to their substantial overlap, dietary fiber was excluded from meta-regression models estimating changes in SBP and LDL-C in which fruits, vegetables, nuts and seeds, and whole grains were independent variables. Likewise, fruits, vegetables, nuts and seeds, and whole grains were excluded in models in which dietary fiber was an independent variable.

Table adapted with permission from Micha R, Shulkin ML, Penalvo JL, et al. Etiologic effects and optimal intakes of foods and nutrients for risk of cardiovascular diseases and diabetes: Systematic reviews and meta-analyses from the Nutrition and Chronic Diseases Expert Group (NutriCoDE). *PLoS One*. 2017;12(4):e0175149.

# **Table G.** Comparison of Relative Risks for CHD Observed in a Large Randomized Clinical Trial of Dietary Patterns vs. Estimated Relative Risks for Individual Dietary Factor. *^a^*

| **Dietary Factor** | **Achieved change in the EVOO group, g/d** *^b^* | **Achieved change in the nut group, g/d** | **NutriCoDE -estimated effect on MI[10]** | **NutriCoDE serving size, g/d** | **Calculated effect on MI in the EVOO group[10]** | **Calculated effect on MI in the nut group[10]** | **Calculated effect on MI in combined groups[10]** |
| --- | --- | --- | --- | --- | --- | --- | --- |
| Fruits | 6.25 | 12.5 | 0.94 | 100 | 0.996 | 0.992 | 0.994 |
| Vegetables | 1.75 | 10.0 | 0.95 | 100 | 0.999 | 0.995 | 0.997 |
| Beans/legumes | 2.40 | 2.4 | 0.77 | 100 | 0.994 | 0.994 | 0.994 |
| Nuts/seeds | 3.25 | 21.0 | 0.77 *^c^* | 16.2 | 0.949 | 0.713 | 0.822 |
| Seafood n-3 fatty acids | 0.11 | 0.12 | 0.92 *^d^* | 0.1 | 0.912 | 0.905 | 0.909 |
| Extra-virgin olive oil (%E) | 4.97 | 1.08 | 0.90 *^e^* | 5.0 | 0.901 | 0.977 | 0.938 |
|  | ***Calculated Overall Effect (all six dietary factors)*** | | | | ***0.771*** | ***0.618*** | ***0.691*** |
|  | ***Observed Effect in PREDIMED*** | | | | ***0.800*** | ***0.740*** | ***0.770*** |

*^a^* For consistency with the other validity analyses (S1 Appendix Tables E-G), we focused on results for CHD in the PREDIMED (Prevencion con Dieta Mediterranea) trial.[32] A similar analysis was previously reported using 2010 estimated RR’s;[33] the findings here are based on the updated RR’s in Micha et al.[5]

*^b^* Values are g/d except for extra virgin olive oil (EVOO), which is percent energy (%E).

*^c^* Assuming half of myocardial infarctions (MIs) were fatal, and half nonfatal.

*^d^* Assuming half of MI’s were fatal, as the risk reduction for marine n-3 fatty acids is specific for fatal MI, not nonfatal MI.

*^e^* Assuming effects on MI are similar to those of vegetable oil polyunsaturated fatty acids.

EVOO, extra-virgin olive oil. NutriCoDE, Nutrition and Chronic Diseases Expert Group.

Table adapted with permission from Micha R, Shulkin ML, Penalvo JL, et al. Etiologic effects and optimal intakes of foods and nutrients for risk of cardiovascular diseases and diabetes: Systematic reviews and meta-analyses from the Nutrition and Chronic Diseases Expert Group (NutriCoDE). *PLoS One*. 2017;12(4):e0175149.

**Table H**. Estimated intervention costs to implement a financial incentive program to promote intake of healthful foods through Medicare and Medicaid.

| **Cost category** | **Cost** | **Source** |
| --- | --- | --- |
| **Policy costs** |  |  |
| Administrative costs^a^, % of total subsidy costs |  |  |
| Year 1 | 20 | SNAP report[22, 34] |
| Year 2 | 5 | CMS report[35] |
| Subsidy costs on dietary target^b^, $ per 100 g |  |  |
| Fruits | 0.34 | USDA Economic Research Service (ERS) Quarterly Food-at-Home Price Database[36] |
| Vegetables | 0.29 |  |
| Whole grains | 0.64 |  |
| Nuts/seeds | 0.76 |  |
| Plant oils | 0.76 |  |
| Seafood | 1.15 |  |

^a^ Administrative costs were derived from SNAP and CMS reports. Administrative costs were assumed to be higher (20% of total subsidy costs) in the first year of implementation based on SNAP administrative costs in 2004 when the EBT system was implemented in all states,[22, 34] and lower (5% of total subsidy costs) in subsequent years based on CMS data demonstrating overall administrative costs of Medicaid to be about 5% of expenditures.[35]

^b^ Average retail costs of foods in each category were estimated using the ERS Quarterly Food-At-Home Price Database version 1[36] (prices of random-weight foods were not included in version 2). We used 2006 data, stratified by region, and calculated the weighted national average price (unit cost) of each dietary component, inflated to constant 2017 dollars.

**Table I**. Estimated formal healthcare, informal healthcare, and productivity costs.^a^

|  | **Cost, $^b^** | **Source** |
| --- | --- | --- |
| **Formal healthcare costs** |  |  |
| **CVD costs:** |  |  |
| **Chronic disease states, per year** |  |  |
| Chronic coronary heart disease | 3,362 | Lee 2010[37] |
| Chronic stroke | 2,222 | Pignone 2006[38] |
| **Acute disease states** |  |  |
| Acute cardiac arrest | 20,242 | O’Sullivan 2011[25] |
| Acute myocardial infarction | 58,254 |  |
| Acute angina | 30,607 |  |
| Acute stroke | 20,092 |  |
| **Procedures and repeat events** |  |  |
| Repeat myocardial infarction | 58,254 | O’Sullivan 2011[25] |
| Repeat stroke | 20,092 |  |
| Coronary artery bypass graft | 38,730 |  |
| Percutaneous coronary intervention | 36,493 |  |
| **Screening** |  |  |
| GP visit in stage 1 | 79 | Pletcher 2009[39] |
| Cholesterol lab test | 37 |  |
| No. of extra GP visits during stage 2 | 1 | assumption |
| No. of lab tests/year after treatment | 1 | Lazar 2011[40] |
| No. of GP visits/year after treatment | 1 |  |
| **Medications, per year** |  |  |
| Statin | 280 | Redbook 2009[41] |
| Anti-hypertensive | 213 | Nuckols 2011[42] |
| Aspirin | 8 | Pignone 2006[38] |
| ACE inhibitor | 54 | Shah 2011[43], Redbook 2009[41] |
| Beta blocker | 54 |  |
| **Statin-associated adverse events** | |  |
| Mild adverse event | 185 | Lee 2010[37] |
| Major adverse event | 7,280 |  |
| **Type 2 diabetes costs^c^** |  |  |
| **Institutional care, per year**  Hospital inpatient  Nursing/residential facility  Hospice  **Outpatient care, per year**  Physician office  Emergency department  Ambulance services  Hospital outpatient  Home health  Podiatry  **Medications and supplies, per year**  Insulin  Diabetic supplies  Other antidiabetic agents  Other prescription medications  Other equipment and supplies | 2,495  485  1  501  219  7  166  147  7  202  76  399  1,043  35 | ADA 2013[44], Zhuo 2013[45] |
| **Informal healthcare costs** |  |  |
| Time per outpatient visit, min |  |  |
| Travel | 35 | Russell 2008[46] |
| Waiting | 42 | Russell 2008[46] |
| Wage for adults aged >45 y, per hour | 15.19 | Bureau of Labor Statistics 2013[47] |
| **Productivity costs** |  |  |
| Labor Force Participation Rates as Full-Time Workers |  | Kim 2016[48] |
| 30 ≤ Age ≤ 44 | 0.845 |  |
| 45 ≤ Age ≤ 59 | 0.826 |  |
| 60 ≤ Age ≤ 64 | 0.566 |  |
| 65 ≤ Age ≤ 69 | 0.321 |  |
| 70 ≤ Age ≤ 74 | 0.195 |  |
| 75 ≤ Age | 0.076 |  |
| Average Annual Earnings |  | Kim 2016[48] |
| 35 ≤ Age ≤ 44 | $53,325 |  |
| 45 ≤ Age ≤ 54 | $54,761 |  |
| 55 ≤ Age ≤ 64 | $55,363 |  |
| 65 ≤ Age ≤ 74 | $42,893 |  |
| 75 ≤ Age | $38,723 |  |

^a^ Formal healthcare costs included costs for all chronic and acute disease states, surgical procedures, screening, treatments, and statin associated adverse effects; diabetes, institutional care, outpatient care, medications and supplies. Informal healthcare costs included costs for patient’s travel and waiting time.

^b^ All costs inflated to constant 2017 dollars using the Bureau of Labor Statistics’ Consumer Price Index.[49]

^c^ Type 2 diabetes costs were derived from analyses by the American Diabetes Association (ADA),[44] modified to exclude costs for complications due to cardiovascular disease (CVD) based on an estimated 53% of type 2 diabetes-related lifetime medical costs being due to treating diabetic complications, of which 57% were due to CVD.[45]

ACE - angiotensin-converting enzyme inhibitor, GP - general practitioner.

# **Table J.** Model Inputs for Probabilistic Sensitivity Analysis.^a^

| **Inputs** | **Mean value (SD)** | **Distribution** | **Source** |
| --- | --- | --- | --- |
| **Unit food costs^b^** |  |  |  |
| Fruits ($/100g) | 0.33680 (0.084) | Gamma |  |
| Vegetables ($/100g) | 0.28901 (0.072) | Gamma | USDA Economic Research Service (ERS) Quarterly Food-at-home Price Database[50] |
| Whole grains ($/100g) | 0.6442 (0.003) | Gamma |  |
| Nuts ($/100g) | 0.7632 (0.191) | Gamma |  |
| Fish ($/100g) | 1.15186 (0.288) | Gamma |  |
| Plant-based oils ($/100g) | 0.75732 (0.189) | Gamma |  |
| **Change in intake of healthful foods for a 30% price change for base case^c^** | Table C |  |  |
| Individuals with PIR <1.3 | 40.5 (8.1) | Normal | Afshin et al. 2017[27] |
| Individuals with PIR ≥1.3 | 34.3 (6.9) | Normal | Afshin et al. 2017[27] |
| **Dietary intake and CVD relative risks** | Table D | Log-normal | Micha et al. 2017[29] |
| **CVD risks** | | | |
| Patients with adverse events, mean % | | | |
| Major | 0.006 (0.0003) | Beta | Zhang et al. 2013[51] |
| Probability major adverse event is fatal, mean % | 0.09 (0.0045) | Beta | Alsheikh-Ali et al. 2005 [52] |
| **CVD healthcare costs, $ 2017** | | | |
| General practitioner screening visit | 79 (7.67) | Gamma | RBRVS[53] |
| Cholesterol laboratory test | 37 (5.83) | Gamma | RBRVS[53] |
| **Acute** | | | |
| Cardiac arrest | 20,242 (963.55) | Gamma | O’Sullivan et al. 2011[25] |
| Fatal myocardial infarction | 18,129 (862.95) | Gamma | O’Sullivan et al. 2011[25] |
| Nonfatal myocardial infarction | 65,334 (3,100) | Gamma | O’Sullivan et al. 2011[25] |
| Angina | 30,607 (1,456) | Gamma | O’Sullivan et al. 2011[25] |
| Fatal stroke | 11,183 (532.35) | Gamma | O’Sullivan et al. 2011[25] |
| Nonfatal stroke | 21,542 (1,025.45) | Gamma | O’Sullivan et al. 2011[25] |
| Coronary artery bypass grafting | 38,730 (1,843.6) | Gamma | O’Sullivan et al. 2011[25] |
| Percutaneous transluminal coronary  angioplasty | 36,493 (1737.1) | Gamma | O’Sullivan et al. 2011[25] |
| Post-first-year annual cost, $ 2017 | | | |
| CHD | 3,362 (533.67) | Gamma | Lee et al., 2010[37] |
| Stroke | 2,222 (352.5) | Gamma | Pignone et al. 2006[38] |
| **Utility weights** |  |  |  |
| Disease free | 1 | Beta | Assumption |
| Cardiac arrest | 0.808 (0.0404) | Beta | Sullivan et al. 2006[21] |
| Myocardial infarction | 0.778 (0.0389) | Beta | Sullivan et al. 2006[21] |
| Angina | 0.768 (0.0384) | Beta | Sullivan et al. 2006[21] |
| Stroke | 0.768 (0.0384) | Beta | Sullivan et al. 2006[21] |

^a^ Because the CVD-PREDICT model reports the average pooled population and stratum effects for each microsimulation, potential variation related to individual-level uncertainty in health state transitions was not incorporated and would have little influence on the pooled average findings. Please note that we performed probabilistic sensitivity analyses from a healthcare perspective. Thus, informal healthcare costs and productivity costs were not included in the table.

^b^ SDs for costs were defined as 25% of the central cost estimate.

^c^ Based on a meta-analysis of intervention and prospective observational studies testing effects of price change on change in intake of healthful foods.

SD - standard deviation; PIR - poverty income ratio; CVD - cardiovascular disease; CHD - coronary heart disease; RBRVS - resource-based relative value scale.

**Table K**. Baseline demographic and health characteristics, and dietary intakes of U.S. adults on Medicare and Medicaid aged 35-80 years.^a^

| **Characteristic** | **Overall^b^** | **Medicare^c^** | **Medicaid^d^** | **Dual-eligible^e^** |
| --- | --- | --- | --- | --- |
| US adults (35-80y) represented, million | 82.0 | 58.2 | 35.2 | 11.4 |
| **Demographics** |  |  |  |  |
| Age, years | 68.1 ± 11.4 | 70.8 ± 8.5 | 54.0 ± 12.7 | 63.2 ± 11.3 |
| Female, % | 56.2 | 54.8 | 64.9 | 64.3 |
| Race/ethnicity, % |  |  |  |  |
| Non-Hispanic white | 74.5 | 80.4 | 40.2 | 49.9 |
| Non-Hispanic black | 11.8 | 9.1 | 27.4 | 22.3 |
| Hispanic | 9.4 | 6.8 | 24.4 | 20.1 |
| Others | 4.4 | 3.8 | 8.0 | 7.7 |
| Education |  |  |  |  |
| <High school | 25.8 | 23.8 | 40.8 | 45.1 |
| High school graduate or some college | 54.7 | 55.1 | 51.4 | 50.3 |
| College graduate | 19.5 | 21.1 | 7.9 | 4.6 |
| Poverty-income ratio^f^ |  |  |  |  |
| <1.30 | 35.0 | 28.9 | 74.2 | 70.7 |
| ≥1.30 | 65.0 | 71.1 | 25.8 | 29.3 |
| SNAP |  |  |  |  |
| Ineligible | 67.4 | 75.2 | 17.9 | 21.6 |
| Eligible non-participants | 13.6 | 13.2 | 16.0 | 15.7 |
| Participants | 19.0 | 11.6 | 66.1 | 62.7 |
| **CVD risk factors** |  |  |  |  |
| Systolic blood pressure, mmHg | 130.3 ± 19.7 | 131.4 ± 19.6 | 125.7 ± 19.3 | 130.7 ± 19.7 |
| Diastolic blood pressure, mmHg | 40.6 ± 35.1 | 40.1 ± 34.8 | 44.9 ± 36.6 | 47.6 ± 36.4 |
| Body mass index, kg/m^2^ | 29.4 ± 6.7 | 29.1 ± 6.5 | 31.4 ± 7.7 | 31.7 ± 7.2 |
| Total cholesterol, mg/dl | 194.4 ± 42.6 | 193.5 ± 42.6 | 197.0 ± 41.4 | 189.9 ± 38.3 |
| HDL cholesterol, mg/dl | 54.6 ± 16.4 | 55.0 ± 16.4 | 52.4 ± 16.3 | 53.8 ± 16.3 |
| Current smoker, % | 14.7 | 11.2 | 32.6 | 19.9 |
| Current hypertension treatment, % | 57.3 | 58.9 | 52.1 | 65.3 |
| **Prevalent disease** |  |  |  |  |
| Diabetes, % | 20.2 | 20.6 | 22.1 | 31.4 |
| Angina, % | 5.9 | 6.4 | 4.6 | 10.0 |
| Myocardial infarction, % | 9.8 | 10.3 | 7.4 | 10.5 |
| Stroke, % | 8.3 | 8.8 | 8.5 | 16.1 |
| CVD free, %^g^ | 81.3 | 80.0 | 83.4 | 67.8 |
| **Dietary intakes** |  |  |  |  |
| Fruits, g/day | 130.3 ± 134.2 | 136.1 ± 135.6 | 101.3 ± 124.7 | 122.7 ± 137.5 |
| Vegetables, g/day | 175.7 ± 133.2 | 178.7 ± 133.9 | 155.7 ± 123.8 | 154.9 ± 118.1 |
| Whole grains, g/day | 24.3 ± 28.0 | 25.6 ± 28.4 | 16.1 ± 22.8 | 15.9 ± 20.8 |
| Nuts/seeds, g/day | 11.9 ± 30.1 | 12.5 ± 30.0 | 7.3 ± 27.5 | 6.0 ± 16.6 |
| Seafood, g/day | 20.6 ± 45.0 | 20.0 ± 44.4 | 24.9 ± 49.0 | 25.8 ± 50.8 |
| Plant oils, g/day | 23.1 ± 13.0 | 23.5 ± 13.1 | 21.3 ± 12.3 | 23.1 ± 13.1 |

^a^ Based on the National Health and Nutrition Examination Survey (NHANES) 2009-2014.[54] All means and percentages were weighted utilizing NHANES sampling and survey weights to represent the national population, with a survey sample size of 26,448 adults on Medicare and Medicaid aged 35-80 y with two 24-hour dietary recalls and reporting Medicare and/or Medicaid insurance coverage. Numbers may not sum to 100 due to rounding.

^b^ Includes Medicare only, Medicaid only, and dual-eligible beneficiaries. Values are mean ± standard deviation. The number of overall population (n=82.0 million) is not equal to the sum of Medicare (n=58.2 million) and Medicaid (n=35.2 million) because dual-eligible (n=11.4 million) is included in both Medicare and Medicaid.

^c^ Includes Medicare only and dual-eligible beneficiaries.

^d^ Includes Medicaid only and dual-eligible beneficiaries.

^e^ Beneficiaries on both Medicare and Medicaid.

^f^ Poverty-income ratio represents the ratio of family income to the federal poverty threshold, adjusting for household size.

^g^ Cardiovascular (CVD) free indicates absence of angina, myocardial infarction (MI), or stroke.

SNAP - Supplementary Nutrition Assistance Program, HDL - high-density lipoprotein cholesterol, LDL - Low-density lipoprotein cholesterol.

**Table L**. Baseline consumption levels and estimated changes in consumption per person at 1 year for individuals on Medicare and/or Medicaid for the two policy scenarios: results of probabilistic sensitivity analyses.^a^

|  | **Baseline Consumption^b^** | | | | **Change in Consumption**^c^ | | | |
| --- | --- | --- | --- | --- | --- | --- | --- | --- |
|  | Overall | Medicare | Medicaid | Dual-eligible | Overall | Medicare | Medicaid | Dual-eligible |
| **Food Categories** |  |  |  |  |  |  |  |  |
| Fruits, g/d | 130.3 | 136.1 | 101.3 | 122.7 | 41.2  (28.4, 53.9) | 43.3  (29.5, 56.9) | 33.9  (23.7, 43.7) | 40.1  (28.3, 50.6) |
| Vegetables, g/d | 175.7 | 178.7 | 155.7 | 154.9 | 43.9  (30.6, 56.2) | 44.0  (30.2, 56.4) | 44.6  (30.9, 58.4) | 43.0  (30.5, 55.9) |
| Whole grains, g/d | 24.3 | 25.6 | 16.1 | 15.9 | 8.1  (5.9, 10.6) | 8.5  (6.3, 11.1) | 5.8  (3.8, 7.8) | 5.6  (3.8, 7.5) |
| Nuts/seeds, g/d | 11.9 | 12.5 | 7.3 | 6.0 | 3.8  (2.6, 5.0) | 4.0  (2.7, 5.3) | 2.7  (1.9, 3.4) | 2.1  (1.6, 2.7) |
| Seafood, g/d | 20.6 | 20.0 | 24.9 | 25.8 | 4.8  (3.4, 6.1) | 4.4  (3.1, 5.7) | 7.3  (5.2, 9.6) | 7.3  (4.8, 9.6) |
| Plant oils, g/d | 23.1 | 23.5 | 21.3 | 23.1 | 6.0  (4.3, 7.6) | 5.9  (4.2, 7.7) | 6.1  (4.2, 7.8) | 6.8  (4.7, 8.8) |

^a^ The two policy scenarios were 1) a 30% financial incentive for purchases of fruits and vegetables (F&V incentive) and 2) a 30% financial incentive for purchases of broader healthy foods including F&V, whole grains, nuts/seeds, seafood, and plant oils (healthy food incentive). See Table B for food category details and definitions. We assumed the time lag between policy implementation and dietary changes occurred within one year, with the intervention effect sustained throughout the simulated period.

^b^ Derived using data from NHANES 2009-2014,[54] based on adults on Medicare and Medicaid with two 24-hour dietary recalls per person.

^c^ Estimated policy effects accounted for the expected change in intake of each food item due to the intervention and the percentage of each food item purchased at stores (e.g., supermarkets, grocery stores, and farmers markets) (see Table C). Values are median estimates (95% UI).

**Table M**. Lifetime health gains, costs, and cost-effectiveness of 30% F&V incentive and healthy food incentive programs through Medicare and Medicaid from a societal perspective.^a^

|  | **Overall^b^** | **Medicare^c^** | **Medicaid^d^** | **Dual-eligible^e^** |
| --- | --- | --- | --- | --- |
| US adults (35-80y) represented, million | 82.0 | 58.2 | 35.2 | 11.4 |
| Simulated years per person^f^ | 18.3 | 16.1 | 29.3 | 21.1 |
| **Scenario 1: F&V incentive (30%)** |  |  |  |  |
| Cases averted, million |  |  |  |  |
| CVD cases | 1.95 | 1.29 | 1.17 | 0.37 |
| CVD deaths | 0.35 | 0.25 | 0.15 | 0.04 |
| Diabetes cases^g^ | -0.006 | -0.003 | -0.01 | -0.003 |
| QALYs gained, million^h^ | 4.72 | 3.19 | 2.45 | 0.64 |
| Change in policy costs, $ billion^i^ |  |  |  |  |
| Administrative costs | 7.21 | 4.97 | 3.55 | 1.04 |
| Food subsidy costs | 117.1 | 79.8 | 61.0 | 17.3 |
| Change in health-related costs, $ billion^j^ |  |  |  |  |
| Formal healthcare costs^k^ | -40.9 | -27.7 | -23.8 | -7.5 |
| Informal healthcare costs^l^ | -0.04 | -0.02 | -0.03 | -0.004 |
| Productivity costs ^m^ | -14.7 | -8.9 | -12.3 | -2.7 |
| Net costs, $ billion^n^ | 68.8 | 48.1 | 28.4 | 8.1 |
| ICER, $/QALY^0^ | 14,576 | 15,113 | 11,589 | 12,668 |
| **Scenario 2: Healthy food incentive (30%)** |  |  |  |  |
| Cases averted, million |  |  |  |  |
| CVD cases | 3.31 | 2.17 | 2.09 | 0.62 |
| CVD deaths | 0.63 | 0.44 | 0.31 | 0.07 |
| Diabetes cases^g^ | 0.12 | 0.08 | 0.07 | 0.01 |
| QALYs gained, million^h^ | 8.48 | 5.68 | 4.75 | 1.16 |
| Change in policy costs, $ billion |  |  |  |  |
| Administrative costs | 12.2 | 8.3 | 6.4 | 1.8 |
| Food subsidy costs | 199.0 | 133.3 | 110.3 | 29.5 |
| Change in health-related costs, $ billion^i^ |  |  |  |  |
| Formal healthcare costs^j^ | -102.4 | -67.6 | -64.1 | -19.2 |
| Informal healthcare costs^k^ | -0.06 | -0.03 | -0.06 | -0.01 |
| Productivity costs ^m^ | -28.3 | -16.8 | -24.4 | -5.3 |
| Net costs, $ billion^n^ | 80.5 | 57.2 | 28.1 | 6.8 |
| ICER, $/QALY^0^ | 9,497 | 10,078 | 5,916 | 5,842 |

^a^ Health outcomes were evaluated among Medicare, Medicaid, and dual-eligible beneficiaries aged 35-80 years at baseline, and followed until death or age 100, whichever came first.

^b^ Includes Medicare only, Medicaid only, and dual-eligible beneficiaries. The number of overall population (n=82 million) is not equal to sum of Medicare (n=58.2 million) and Medicaid (n=35.2 million) because dual-eligible (n=11.4 million) is included in both Medicare and Medicaid.

^c^ Includes Medicare only and dual-eligible beneficiaries.

^d^ Includes Medicaid only and dual-eligible beneficiaries.

^e^ Beneficiaries on both Medicare and Medicaid.

^f^ The average number of years for all simulated individuals in the model.

^g^ We did not identify probable or convincing evidence of etiologic effects of fruits and vegetables on diabetes, the *F&V incentive* resulted in a slightly higher number of diabetes cases compared to a base-case of no new intervention due to increased overall survival from prevented cardiovascular disease (CVD).

^h^ Quality-adjusted life years (QALYs) were discounted at 3% annually.

^i^ Policy costs included total administrative costs and food subsidy costs. All costs were inflated in 2017 dollars.

^j^ Negative costs indicate health-related savings.

^k^ Formal healthcare costs were calculated from the change in total healthcare costs associated with CVD events including chronic/acute disease states, surgical procedures, screening costs, and drug costs; with diabetes cases including institutional care, outpatient care, outpatient medications and supplies, discounted at 3% annually.

^l^ Informal health costs were calculated from change in costs associated with patient’s travel and waiting time as derived from the Bureau of Labor Statistics (Table I), discounted at 3% annually.

^m^ Productivity costs were calculated from the change in costs associated with productivity, accounting for age-specific average annual earnings and labor force participation rates (Table I), discounted at 3% annually.

^n^ Net costs from a societal perspective = Policy costs – health-related savings (including formal/informal healthcare costs and indirect costs), discounted at 3% annually.

^o^ According to the ACC/AHA, ICERs below $50,000/QALY and at $50,000-$150,000/QALY are considered highly cost effective and cost-effective, respectively.[55]

QALY - quality-adjusted life-year, ICER - incremental cost-effectiveness ratio.

**Table N.** Results of probabilistic sensitivity analyses at 5 years.^a^

|  | **Median Estimate (95% UI)** | | | |
| --- | --- | --- | --- | --- |
|  | **Overall^b^** | **Medicare^c^** | **Medicaid^d^** | **Dual-eligible^e^** |
| US adults (35-80y) represented, million | 82.0 | 58.2 | 35.2 | 11.4 |
| **Scenario 1: F&V incentive (30%)** |  |  |  |  |
| Cases averted, million |  |  |  |  |
| CVD cases | 0.51 (0.42, 0.61) | 0.39 (0.31, 0.47) | 0.18 (0.15, 0.21) | 0.09 (0.07, 0.11) |
| CVD deaths | 0.05 (0.04, 0.06) | 0.04 (0.03, 0.05) | 0.013 (0.011, 0.016) | 0.005 (0.004, 0.007) |
| Diabetes cases^f^ | -0.0023  (-0.0024, -0.0021) | -0.0003  (-0.0007, -0.0001) | 0.0006  (0.0005, 0.0006) | -0.0003  (-0.0003, -0.0003) |
| QALYs gained, million^g^ | 0.27 (0.20, 0.35) | 0.20 (0.15, 0.27) | 0.08 (0.06, 0.10) | 0.03 (0.02, 0.04) |
| Change in policy costs, $ billion^h^ |  |  |  |  |
| Administrative costs | 3.33 (2.34, 4.59) | 2.39 (1.65, 3.27) | 1.31 (0.92, 1.82) | 0.53 (0.37, 0.73) |
| Food subsidy costs | 39.9 (28.0, 55.0) | 28.6 (19.7, 39.1) | 15.9 (11.2, 22.1) | 6.37 (4.42, 8.77) |
| Change in formal healthcare cost, $ billion^i^ | -14.6 (-17.7, -11.6) | -11.1 (-13.3, -8.79) | -5.38 (-6.54, -4.27) | -3.10 (-3.84, -2.39) |
| Net costs, healthcare perspective, $ billion^j^ | 28.8 (15.5, 44.6) | 20.5 (10.6, 31.7) | 11.4 (6.33, 17.4) | 3.18 (1.23, 5.41) |
| ICER, $/QALY^k^ | 107,343  (54,385, 183,068) | 100,636  (49,606, 167,388) | 148,419  (77,734, 245,009) | 103,790  (41,227, 190,401) |
| **Scenario 2: Healthy food incentive (30%)** |  |  |  |  |
| Cases averted, million |  |  |  |  |
| CVD cases | 0.90 (0.79, 1.02) | 0.67 (0.58, 0.77) | 0.31 (0.28, 0.35) | 0.15 (0.13, 0.17) |
| CVD deaths | 0.10 (0.09, 0.11) | 0.08 (0.07, 0.09) | 0.03 (0.02, 0.03) | 0.010 (0.009, 0.012) |
| Diabetes cases^f^ | 0.04 (0.03, 0.05) | 0.03 (0.02, 0.04) | 0.02 (0.01, 0.02) | 0.004 (0.003, 0.004) |
| QALYs gained, million^g^ | 0.47 (0.38, 0.57) | 0.35 (0.29, 0.43) | 0.13 (0.11, 0.15) | 0.05 (0.04, 0.06) |
| Change in policy costs, $ billion^h^ |  |  |  |  |
| Administrative costs | 5.66 (4.47, 7.01) | 4.03 (3.19, 4.99) | 2.35 (1.86, 2.93) | 0.86 (0.68, 1.08) |
| Food subsidy costs | 67.9 (53.7, 84.1) | 48.3 (38.2, 59.7) | 28.5 (22.6, 35.5) | 10.4 (8.19, 13.0) |
| Change in formal healthcare cost, $ billion^i^ | -33.2 (-37.2, -28.8) | -24.7 (-28.1, -21.5) | -13.2 (-15.0, -11.4) | -7.19 (-8.29, -6.06) |
| Net costs, healthcare perspective, $ billion^j^ | 40.9 (25.3, 57.9) | 28.2 (16.6, 40.9) | 17.7 (11.4, 24.9) | 4.06 (1.61, 6.58) |
| ICER, $/QALY^k^ | 86,550 (51,187, 130,695) | 79,391 (44,973, 120,905) | 136,700 (83,501, 199,603) | 80,273 (33,038, 135,705) |

^a^ Health outcomes were evaluated among Medicare, Medicaid, and dual-eligible beneficiaries aged 35-80 years at baseline, and followed until death or age 100, whichever came first.

^b^ Includes Medicare only, Medicaid only, and dual-eligible beneficiaries. The number of overall population (n=82 million) is not equal to sum of Medicare (n=58.2 million) and Medicaid (n=35.2 million) because dual-eligible (n=11.4 million) is included in both Medicare and Medicaid.

^c^ Includes Medicare only and dual-eligible beneficiaries.

^d^ Includes Medicaid only and dual-eligible beneficiaries.

^e^ Beneficiaries on both Medicare and Medicaid.

^f^ We did not identify probable or convincing evidence of etiologic effects of fruits and vegetables on diabetes, the *F&V incentive* resulted in a slightly higher number of diabetes cases compared to a base-case of no new intervention due to increased overall survival from prevented cardiovascular disease (CVD).

^g^ Quality-adjusted life years (QALYs) were discounted at 3% annually.

^h^ Policy costs included total administrative costs and food subsidy costs. All costs were inflated in 2017 dollars.

^i^ Negative costs indicate health-related savings. Formal healthcare costs were calculated from the change in total healthcare costs associated with CVD events including chronic/acute disease states, surgical procedures, screening costs, and drug costs; with diabetes cases including institutional care, outpatient care, outpatient medications and supplies, discounted at 3% annually.

^j^ Net costs from a healthcare perspective = Policy costs – formal healthcare savings, discounted at 3% annually.

^k^ According to the ACC/AHA, ICERs below $50,000/QALY and at $50,000-$150,000/QALY are considered highly cost effective and cost-effective, respectively.[55]

QALY - quality-adjusted life-year, ICER - incremental cost-effectiveness ratio.

**Table O**. Lifetime estimated health gains, costs, and cost-effectiveness of the 30% F&V and health food incentive programs, by race/ethnicity, education, Supplemental Nutrition Assistance Program, income and age.^a^

|  | Cases averted | | | | QALYs  gained | Healthcare perspective^b^ | | Societal perspective^b^ | |
| --- | --- | --- | --- | --- | --- | --- | --- | --- | --- |
|  | | CVD cases averted | CVD deaths averted | Diabetes cases averted^c^ |  | Net costs^d^, $ billion | ICER^e^, $/QALY | Net costs^f^, $ billion | ICER^e^, $/QALY |
| **Scenario 1: F&V incentive (30%)** | | | | | | | |  |  |
| **Medicare** | |  |  |  |  |  |  |  |  |
| Non-Hispanic White | | 988,356 | 190,358 | -2,151 | 2,519,640 | 45.77 | 18,166 | 38.57 | 15,308 |
| Non-Hispanic Black | | 132,306 | 19,081 | -89 | 281,229 | 4.52 | 16,067 | 3.64 | 12,927 |
| Hispanic | | 112,629 | 16,607 | -590 | 242,306 | 4.01 | 16,550 | 3.27 | 13,508 |
| Others | | 72,555 | 12,709 | -71 | 183,236 | 2.97 | 16,234 | 2.42 | 13,196 |
| <High school | | 299,754 | 41,730 | -982 | 600,008 | 9.38 | 15,625 | 7.73 | 12,876 |
| High school or some college | | 725,719 | 128,295 | -3,048 | 1,786,726 | 30.83 | 17,255 | 25.50 | 14,271 |
| College graduate or above | | 275,180 | 59,545 | -1,203 | 804,142 | 12.03 | 14,954 | 9.91 | 12,318 |
| SNAP participants | | 182,418 | 21,222 | -1,053 | 335,884 | 5.57 | 16,573 | 4.35 | 12,936 |
| SNAP eligible, non-participants | | 177,069 | 25,448 | -223 | 386,304 | 5.29 | 13,690 | 4.21 | 10,895 |
| SNAP ineligible individuals | | 939,253 | 186,136 | -1,006 | 2,468,953 | 46.41 | 18,799 | 39.75 | 16,102 |
| Low income^g^ | | 384,034 | 62,180 | -2,268 | 856,618 | 12.84 | 14,991 | 10.15 | 11,847 |
| High income^g^ | | 908,472 | 182,125 | -2,773 | 2,363,485 | 44.49 | 18,821 | 38.05 | 16,098 |
| **Medicaid** | |  |  |  |  |  |  |  |  |
| Non-Hispanic White | | 410,130 | 48,003 | -3,622 | 840,707 | 12.73 | 15,146 | 8.25 | 9,819 |
| Non-Hispanic Black | | 388,204 | 46,705 | -3,109 | 788,638 | 12.44 | 15,777 | 8.35 | 10,585 |
| Hispanic | | 284,333 | 44,891 | -1,869 | 628,246 | 12.07 | 19,205 | 9.24 | 14,707 |
| Others | | 95,676 | 11,719 | -883 | 211,653 | 3.35 | 15,851 | 2.28 | 10,783 |
| <High school | | 441,785 | 58,890 | -4,581 | 994,253 | 15.38 | 15,469 | 10.65 | 10,709 |
| High school or some college | | 664,898 | 72,367 | -3,904 | 1,266,214 | 20.50 | 16,194 | 13.78 | 10,881 |
| College graduate or above | | 66,177 | 13,077 | -147 | 167,678 | 4.57 | 27,271 | 3.91 | 23,321 |
| SNAP participants | | 783,605 | 93,884 | -4,119 | 1,542,546 | 26.07 | 16,901 | 18.15 | 11,767 |
| SNAP eligible, non-participants | | 196,194 | 27,450 | -1,182 | 422,311 | 6.47 | 15,312 | 4.43 | 10,485 |
| SNAP ineligible individuals | | 194,986 | 28,872 | -2,115 | 470,377 | 7.93 | 16,850 | 5.64 | 11,981 |
| Age (35-54y) | | 745,314 | 103,559 | -3,879 | 1,615,813 | 27.05 | 16,743 | 17.72 | 10,967 |
| Age (55-74y) | | 376,553 | 44,556 | -839 | 729,891 | 11.59 | 15,873 | 9.20 | 12,610 |
| Age (75y+) | | 51,428 | 6,651 | -56 | 84,487 | 1.65 | 19,532 | 1.45 | 17,180 |
| **Dual-eligible** | |  |  |  |  |  |  |  |  |
| Non-Hispanic White | | 159,910 | 17,775 | -2,020 | 337,141 | 5.13 | 15,222 | 3.42 | 10,152 |
| Non-Hispanic Black | | 100,627 | 9,764 | -581 | 148,368 | 2.27 | 15,308 | 1.70 | 11,454 |
| Hispanic | | 81,262 | 8,092 | -110 | 121,874 | 2.19 | 17,990 | 1.84 | 15,108 |
| Others | | 30,663 | 2,508 | -43 | 42,372 | 0.92 | 21,713 | 0.79 | 18,756 |
| <High school | | 137,984 | 17,635 | -1,075 | 260,441 | 4.10 | 15,739 | 3.28 | 12,589 |
| High school or some college | | 223,658 | 18,953 | -1,782 | 357,112 | 5.78 | 16,185 | 3.91 | 10,939 |
| College graduate or above | | 10,438 | 2,559 | -78 | 35,536 | 0.66 | 18,699 | 0.57 | 16,022 |
| SNAP participants | | 216,374 | 20,672 | 364 | 330,125 | 5.54 | 16,792 | 4.36 | 13,214 |
| SNAP eligible, non-participants | | 55,554 | 6,427 | -117 | 93,971 | 1.35 | 14,347 | 1.04 | 11,075 |
| SNAP ineligible individuals | | 102,204 | 12,716 | -1,268 | 223,610 | 3.47 | 15,504 | 2.27 | 10,152 |
| **Scenario 2: Healthy food incentive (30%)** | | | | | | | | | |
| **Medicare** | |  |  |  |  |  |  |  |  |
| Non-Hispanic White | | 1,661,365 | 348,312 | 66,866 | 4,494,381 | 59.97 | 13,342 | 46.53 | 10,352 |
| Non-Hispanic Black | | 224,135 | 34,029 | 4,885 | 487,805 | 5.99 | 12,275 | 4.41 | 9,040 |
| Hispanic | | 181,174 | 28,611 | 5,217 | 414,491 | 4.51 | 10,879 | 3.17 | 7,650 |
| Others | | 121,286 | 21,493 | 3,376 | 309,833 | 3.42 | 11,047 | 2.46 | 7,924 |
| <High school | | 467,793 | 73,075 | 7,854 | 1,028,824 | 14.52 | 14,116 | 11.47 | 11,147 |
| High school or some college | | 1,212,141 | 225,181 | 43,663 | 3,092,068 | 38.60 | 12,482 | 28.87 | 9,336 |
| College graduate or above | | 500,725 | 119,238 | 23,897 | 1,518,331 | 14.12 | 9,303 | 9.99 | 6,582 |
| SNAP participants | | 286,633 | 37,303 | 7,396 | 575,364 | 7.10 | 12,337 | 4.93 | 8,563 |
| SNAP eligible, non-participants | | 301,533 | 48,388 | 8,934 | 701,042 | 7.54 | 10,755 | 5.44 | 7,764 |
| SNAP ineligible individuals | | 1,588,695 | 340,644 | 64,087 | 4,380,013 | 59.26 | 13,530 | 46.62 | 10,643 |
| Low income^g^ | | 635,443 | 106,859 | 17,368 | 1,518,398 | 16.96 | 11,172 | 12.03 | 7,925 |
| High income^g^ | | 1,543,261 | 331,385 | 63,827 | 4,226,127 | 56.89 | 13,459 | 44.71 | 10,578 |
| **Medicaid** | |  |  |  |  |  |  |  |  |
| Non-Hispanic White | | 787,522 | 112,418 | 24,150 | 1,820,477 | 13.06 | 7,176 | 3.13 | 1,722 |
| Non-Hispanic Black | | 660,876 | 95,776 | 30,329 | 1,452,320 | 17.89 | 12,320 | 10.61 | 7,305 |
| Hispanic | | 469,651 | 76,193 | 12,106 | 1,086,818 | 15.52 | 14,277 | 10.24 | 9,425 |
| Others | | 175,414 | 25,481 | 2,450 | 415,181 | 5.80 | 13,979 | 3.67 | 8,838 |
| <High school | | 744,336 | 110,112 | 13,728 | 1,782,160 | 20.13 | 11,297 | 11.06 | 6,207 |
| High school or some college | | 1,224,191 | 165,429 | 36,328 | 2,632,647 | 24.59 | 9,341 | 10.50 | 3,990 |
| College graduate or above | | 119,055 | 26,810 | 18,924 | 311,876 | 7.42 | 23,802 | 6.19 | 19,854 |
| SNAP participants | | 1,373,461 | 192,003 | 46,523 | 2,915,449 | 37.12 | 12,731 | 22.21 | 7,617 |
| SNAP eligible, non-participants | | 357,241 | 57,986 | 15,473 | 865,456 | 7.18 | 8,291 | 2.86 | 3,300 |
| SNAP ineligible individuals | | 361,931 | 57,550 | 9,872 | 939,703 | 7.78 | 8,275 | 2.89 | 3,075 |
| Age (35-54y) | | 1,356,943 | 216,645 | 58,562 | 3,205,814 | 37.58 | 11,722 | 18.62 | 5,808 |
| Age (55-74y) | | 645,794 | 84,987 | 12,602 | 1,407,919 | 12.16 | 8,635 | 7.22 | 5,129 |
| Age (75y+) | | 80,465 | 10,804 | 638 | 134,545 | 2.85 | 21,145 | 2.52 | 18,710 |
| **Dual-eligible** | |  |  |  |  |  |  |  |  |
| Non-Hispanic White | | 278,624 | 37,450 | 6,118 | 674,361 | 5.39 | 7,996 | 1.78 | 2,644 |
| Non-Hispanic Black | | 149,906 | 14,822 | 1,119 | 222,727 | 3.58 | 16,071 | 2.71 | 12,154 |
| Hispanic | | 144,497 | 12,813 | 3,600 | 196,364 | 1.94 | 9,897 | 1.34 | 6,826 |
| Others | | 50,979 | 4,941 | 308 | 75,124 | 1.03 | 13,672 | 0.79 | 10,553 |
| <High school | | 220,161 | 26,144 | 1,806 | 392,449 | 5.93 | 15,099 | 4.67 | 11,892 |
| High school or some college | | 388,423 | 40,909 | 9,139 | 722,722 | 5.21 | 7,204 | 1.30 | 1,795 |
| College graduate or above | | 14,985 | 3,948 | 55 | 52,096 | 0.80 | 15,342 | 0.66 | 12,644 |
| SNAP participants | | -331832 | -34772 | -4329 | 545,164 | 7.66 | 14,056 | 5.68 | 10,420 |
| SNAP eligible, non-participants | | -89290 | -10624 | -1245 | 151,895 | 2.49 | 16,365 | 2.00 | 13,146 |
| SNAP ineligible individuals | | -202189 | -26238 | -7216 | 460,149 | 1.65 | 3,582 | -1.04 | saving |

^a^ The distribution of the population in each subgroup was derived from the survey weighted percentages among adults on Medicare and/or Medicaid in NHANES 2009-2014.[26]

^b^ Net costs and incremental cost-effectiveness ratio (ICER) were evaluated from healthcare and societal perspectives. The healthcare perspective included policy costs and formal healthcare costs. The societal perspective included policy costs, formal/informal healthcare costs, and indirect costs.

^c^ Because we did not identify probable or convincing evidence of etiologic effects of fruits and vegetables on type 2 diabetes (see Table D), the F&V incentive resulted in slightly higher number of cases due to increased overall survival from prevented cardiovascular disease.

^d^ Net costs from a healthcare perspective = Policy costs – formal healthcare savings, discounted at 3% annually.

^e^ According to the ACC/AHA, ICERs below $50,000/QALY and at $50,000-$150,000/QALY are considered highly cost effective and cost-effective, respectively.

^f^ Net costs from a societal perspective = Policy costs – health-related savings (including formal/informal healthcare costs and indirect costs), discounted at 3% annually.

^g^ Defined by income eligibility threshold for government food assistance programs (poverty-income ratio of 1.3).

CVD - cardiovascular disease, QALY - quality-adjusted life year, ICER - incremental cost-effectiveness ratio, SNAP – Supplemental Nutrition Assistance Program

**Table P**. Lifetime health gains, costs, and cost-effectiveness of 30% F&V incentive and healthy food incentive programs among Medicare beneficiaries by age.^a^

|  | **Medicare < 65y**^b^ | **Medicare ≥ 65y**^c^ |
| --- | --- | --- |
| US adults (35-80y) represented, million | 9.08 | 49.1 |
| **Scenario 1: F&V incentive (30%)** |  |  |
| Simulated years per person, years^d^ | 27.7 | 14.2 |
| Cases averted, million |  |  |
| CVD cases | 0.33 | 0.41 |
| CVD deaths | 0.04 | 0.10 |
| Diabetes cases^e^ | -0.003 | -0.017 |
| QALYs gained, million^f^ | 0.64 | 1.08 |
| Change in policy costs, $ billion^g^ |  |  |
| Administrative costs | 0.94 | 4.06 |
| Food subsidy costs | 16.2 | 64.3 |
| Change in formal healthcare cost, $ billion^h^ | -7.0 | -21.3 |
| Net costs, healthcare perspective, $ billion^i^ | 10.1 | 47.0 |
| ICER, $/QALY^j^ | 15,856 | 43,429 |
| **Scenario 2: Healthy food incentive (30%)** | | |
| Simulated years per person, years^d^ | 27.8 | 14.3 |
| Cases averted, million |  |  |
| CVD cases | 0.57 | 1.16 |
| CVD deaths | 0.08 | 0.26 |
| Diabetes cases^e^ | 0.02 | 0.03 |
| QALYs gained, million^f^ | 1.23 | 3.14 |
| Change in policy costs, $ billion^g^ |  |  |
| Administrative costs | 1.55 | 6.79 |
| Food subsidy costs | 26.6 | 107.6 |
| Change in formal healthcare cost, $ billion^h^ | -18.2 | -51.6 |
| Net costs, healthcare perspective, $ billion^i^ | 9.97 | 62.7 |
| ICER, $/QALY^j^ | 8,119 | 19,991 |

^a^ Health outcomes were evaluated among Medicare beneficiaries aged 35-64 years or 65-80 years at baseline, and followed until death or age 100, whichever came first.

^b^ Includes Medicare only and dual-eligible beneficiaries aged 35-64 years (n=9.08 million).

^c^ Includes Medicare only and dual-eligible beneficiaries aged 65-80 years (n=49.1 million).

^d^ The average number of years for all simulated individuals in the model.

^e^ We did not identify probable or convincing evidence of etiologic effects of fruits and vegetables on diabetes, the *F&V incentive* resulted in a slightly higher number of diabetes cases compared to a base-case of no new intervention due to increased overall survival from prevented cardiovascular disease (CVD).

^f^ Quality-adjusted life years (QALYs) were discounted at 3% annually.

^g^ Policy costs included total administrative costs and food subsidy costs. All costs were inflated in 2017 dollars.

^h^ Negative costs indicate health-related savings. Formal healthcare costs were calculated from the change in total healthcare costs associated with CVD events including chronic/acute disease states, surgical procedures, screening costs, and drug costs; with diabetes cases including institutional care, outpatient care, outpatient medications and supplies, discounted at 3% annually.

^i^ Net costs from a healthcare perspective = Policy costs – formal healthcare savings, discounted at 3% annually.

^j^ According to the ACC/AHA, ICERs below $50,000/QALY and at $50,000-$150,000/QALY are considered highly cost effective and cost-effective, respectively.[55]

QALY - quality-adjusted life-year, ICER - incremental cost-effectiveness ratio.

**Table Q**. Lifetime health gains per million persons of 30% F&V incentive and healthy food incentive programs among Medicare beneficiaries by age group.^a^

|  | **Medicare < 65y**^b^ | **Medicare ≥ 65y**^c^ |
| --- | --- | --- |
| US adults (35-80y) represented, million | 9.08 | 49.1 |
| **Scenario 1: F&V incentive (30%)** |  |  |
| Cases averted per million persons |  |  |
| CVD cases | 36,163 | 8,346 |
| CVD deaths | 4,209 | 1,966 |
| Diabetes cases^d^ | -280 | -356 |
| QALYs gained per million persons | 70,371 | 22,052 |
| **Scenario 2: Healthy food incentive (30%)** | | |
| Cases averted per million persons |  |  |
| CVD cases | 62,346 | 23,682 |
| CVD deaths | 8,556 | 5,324 |
| Diabetes cases^d^ | 2,663 | 706 |
| QALYs gained per million persons | 135,339 | 63,910 |

^a^ Health outcomes were evaluated among Medicare beneficiaries aged 35-64 years or 65-80 years at baseline, and followed until death or age 100, whichever came first.

^b^ Includes Medicare only and dual-eligible beneficiaries aged 35-64 years (n=9.08 million).

^c^ Includes Medicare only and dual-eligible beneficiaries aged 65-80 years (n=49.1 million).

^d^ We did not identify probable or convincing evidence of etiologic effects of fruits and vegetables on diabetes, the *F&V incentive* resulted in a slightly higher number of diabetes cases compared to a base-case of no new intervention due to increased overall survival from prevented cardiovascular disease (CVD).

**Table R**. Sensitivity analyses of lifetime health gains, costs, and cost-effectiveness of a 30% healthy food incentive program excluding seafood and plant oils through Medicare and Medicaid.^a^

|  | **Overall^b^** | **Medicare^c^** | **Medicaid^d^** | **Dual-eligible^e^** |
| --- | --- | --- | --- | --- |
| US adults (35-80y) represented, million | 82.0 | 58.2 | 35.2 | 11.4 |
| **Healthy food incentive excluding seafood and plant oils (30%)** |  |  |  |  |
| Simulated years per person, years^f^ | 18.23 | 16.05 | 29.21 | 21.10 |
| Cases averted, million |  |  |  |  |
| CVD cases | 2.83 | 1.90 | 1.64 | 0.52 |
| CVD deaths | 0.53 | 0.38 | 0.23 | 0.06 |
| Diabetes cases^g^ | 0.13 | 0.08 | 0.07 | 0.01 |
| QALYs gained, million^h^ | 7.15 | 4.88 | 3.65 | 0.98 |
| Change in policy costs, $ billion^i^ |  |  |  |  |
| Administrative costs | 9.41 | 6.54 | 4.41 | 1.25 |
| Food subsidy costs | 152.9 | 105.0 | 75.7 | 20.6 |
| Change in health-related costs, $ billion^j^ |  |  |  |  |
| Formal healthcare costs^k^ | -82.2 | -55.9 | -46.4 | -14.8 |
| Informal healthcare costs^l^ | -0.05 | -0.03 | -0.04 | -0.01 |
| Productivity costs ^m^ | -23.4 | -14.3 | -18.4 | -4.5 |
| Net costs, healthcare perspective, $ billion^n^ | 80.1 | 55.6 | 33.8 | 7.1 |
| Net costs, societal perspective, $ billion^o^ | 56.7 | 41.3 | 15.3 | 2.5 |
| ICER, $/QALY^p^ |  |  |  |  |
| Healthcare perspective | 11,201 | 11,388 | 9,239 | 7,206 |
| Societal perspective | 7,920 | 8,449 | 4,181 | 2,584 |

^a^ Health outcomes were evaluated among Medicare, Medicaid, and dual-eligible beneficiaries aged 35-80 years at baseline, and followed until death or age 100, whichever came first.

^b^ Includes Medicare only, Medicaid only, and dual-eligible beneficiaries. The number of overall population (n=82 million) is not equal to sum of Medicare (n=58.2 million) and Medicaid (n=35.2 million) because dual-eligible (n=11.4 million) is included in both Medicare and Medicaid.

^c^ Includes Medicare only and dual-eligible beneficiaries.

^d^ Includes Medicaid only and dual-eligible beneficiaries.

^e^ Beneficiaries on both Medicare and Medicaid.

^f^ The average number of years for all simulated individuals in the model.

^g^ We did not identify probable or convincing evidence of etiologic effects of fruits and vegetables on diabetes, the *F&V incentive* resulted in a slightly higher number of diabetes cases compared to a base-case of no new intervention due to increased overall survival from prevented cardiovascular disease (CVD).

^h^ Quality-adjusted life years (QALYs) were discounted at 3% annually.

^i^ Policy costs included total administrative costs and food subsidy costs. All costs were inflated in 2017 dollars.

^j^ Negative costs indicate health-related savings.

^k^ Formal healthcare costs were calculated from the change in total healthcare costs associated with CVD events including chronic/acute disease states, surgical procedures, screening costs, and drug costs; with diabetes cases including institutional care, outpatient care, outpatient medications and supplies, discounted at 3% annually.

^l^ Informal health costs were calculated from change in costs associated with patient’s travel and waiting time as derived from the Bureau of Labor Statistics (Table I), discounted at 3% annually.

^m^ Productivity costs were calculated from the change in costs associated with productivity, accounting for age-specific average annual earnings and labor force participation rates (Table I), discounted at 3% annually.

^n^ Net costs from a healthcare perspective = Policy costs – formal healthcare savings, discounted at 3% annually.

^o^Net costs from a societal perspective = Policy costs – health-related savings (including formal/informal healthcare costs and indirect costs), discounted at 3% annually.

^p^ According to the ACC/AHA, ICERs below $50,000/QALY and at $50,000-$150,000/QALY are considered highly cost effective and cost-effective, respectively.[55]

QALY - quality-adjusted life-year, ICER - incremental cost-effectiveness ratio.

**Table S**. Lifetime health gains, costs, and cost-effectiveness of 20% F&V incentive and healthy food incentive programs through Medicare and Medicaid.^a^

|  | **Overall^b^** | **Medicare^c^** | **Medicaid^d^** | **Dual-eligible^e^** |
| --- | --- | --- | --- | --- |
| US adults (35-80y) represented, million | 82.0 | 58.2 | 35.2 | 11.4 |
| **Scenario 1: F&V incentive (20%)** |  |  |  |  |
| Simulated years per person, years^f^ | 18.16 | 15.99 | 29.11 | 21.02 |
| Cases averted, million |  |  |  |  |
| CVD cases | 1.32 | 0.87 | 0.79 | 0.25 |
| CVD deaths | 0.24 | 0.17 | 0.10 | 0.02 |
| Diabetes cases^g^ | -0.005 | -0.002 | -0.008 | -0.003 |
| QALYs gained, million^h^ | 3.14 | 2.17 | 1.66 | 0.43 |
| Change in policy costs, $ billion^i^ |  |  |  |  |
| Administrative costs | 4.37 | 3.02 | 2.14 | 0.63 |
| Food subsidy costs | 71.0 | 48.4 | 36.8 | 10.4 |
| Change in formal healthcare cost, $ billion^j^ | -27.6 | -18.6 | -15.8 | -5.0 |
| Net costs, healthcare perspective, $ billion^k^ | 47.8 | 32.9 | 23.2 | 6.0 |
| ICER, $/QALY^l^ | 15,203 | 15,118 | 13,940 | 14,009 |
| **Scenario 2: Healthy food incentive (20%)** |  |  |  |  |
| Simulated years per person, years^f^ | 18.21 | 16.03 | 29.19 | 21.07 |
| Cases averted, million |  |  |  |  |
| CVD cases | 2.27 | 1.48 | 1.44 | 0.43 |
| CVD deaths | 0.43 | 0.30 | 0.21 | 0.05 |
| Diabetes cases^g^ | 0.09 | 0.05 | 0.04 | 0.007 |
| QALYs gained, million^h^ | 5.78 | 3.86 | 3.26 | 0.78 |
| Change in policy costs, $ billion^i^ |  |  |  |  |
| Administrative costs | 7.41 | 5.04 | 3.87 | 1.08 |
| Food subsidy costs | 120.5 | 80.8 | 66.5 | 17.8 |
| Change in formal healthcare cost, $ billion^j^ | -70.8 | -46.5 | -43.9 | -13.1 |
| Net costs, healthcare perspective, $ billion^k^ | 57.1 | 39.3 | 26.5 | 5.8 |
| ICER, $/QALY^l^ | 9,870 | 10,205 | 8,134 | 7,348 |

^a^ Health outcomes were evaluated among Medicare, Medicaid, and dual-eligible beneficiaries aged 35-80 years at baseline, and followed until death or age 100, whichever came first.

^b^ Includes Medicare only, Medicaid only, and dual-eligible beneficiaries. The number of overall population (n=82 million) is not equal to sum of Medicare (n=58.2 million) and Medicaid (n=35.2 million) because dual-eligible (n=11.4 million) is included in both Medicare and Medicaid.

^c^ Includes Medicare only and dual-eligible beneficiaries.

^d^ Includes Medicaid only and dual-eligible beneficiaries.

^e^ Beneficiaries on both Medicare and Medicaid.

^f^ The average number of years for all simulated individuals in the model.

^g^ We did not identify probable or convincing evidence of etiologic effects of fruits and vegetables on diabetes, the *F&V incentive* resulted in a slightly higher number of diabetes cases compared to a base-case of no new intervention due to increased overall survival from prevented cardiovascular disease (CVD).

^h^ Quality-adjusted life years (QALYs) were discounted at 3% annually.

^i^ Policy costs included total administrative costs and food subsidy costs. All costs were inflated in 2017 dollars.

^j^ Negative costs indicate health-related savings. Formal healthcare costs were calculated from the change in total healthcare costs associated with CVD events including chronic/acute disease states, surgical procedures, screening costs, and drug costs; with diabetes cases including institutional care, outpatient care, outpatient medications and supplies, discounted at 3% annually.

^k^ Net costs from a healthcare perspective = Policy costs – formal healthcare savings, discounted at 3% annually.

^l^ According to the ACC/AHA, ICERs below $50,000/QALY and at $50,000-$150,000/QALY are considered highly cost effective and cost-effective, respectively.[55]

QALY - quality-adjusted life-year, ICER - incremental cost-effectiveness ratio.

**Table T.** Lifetime health gains, costs, and cost-effectiveness of 50% F&V incentive and healthy food incentive programs through Medicare and Medicaid.^a^

|  | **Overall^b^** | **Medicare^c^** | **Medicaid^d^** | **Dual-eligible^e^** |
| --- | --- | --- | --- | --- |
| US adults (35-80y) represented, million | 82.0 | 58.2 | 35.2 | 11.4 |
| **Scenario 1: F&V incentive (50%)** |  |  |  |  |
| Simulated years per person, years^f^ | 18.24 | 16.06 | 29.22 | 21.11 |
| Cases averted, million |  |  |  |  |
| CVD cases | 3.13 | 2.09 | 1.86 | 0.59 |
| CVD deaths | 0.56 | 0.40 | 0.45 | 0.06 |
| Diabetes cases^g^ | -0.011 | -0.006 | -0.015 | -0.004 |
| QALYs gained, million^h^ | 7.58 | 5.19 | 3.93 | 1.04 |
| Change in policy costs, $ billion^i^ |  |  |  |  |
| Administrative costs | 14.2 | 9.8 | 7.0 | 2.1 |
| Food subsidy costs | 230.6 | 156.8 | 120.8 | 34.1 |
| Change in formal healthcare cost, $ billion^j^ | -66.2 | -44.8 | -38.3 | -12.3 |
| Net costs, healthcare perspective, $ billion^k^ | 178.6 | 121.8 | 89.5 | 23.9 |
| ICER, $/QALY^l^ | 23,572 | 23,488 | 22,752 | 22,947 |
| **Scenario 2: Healthy food incentive (50%)** |  |  |  |  |
| Simulated years per person, years^f^ | 18.35 | 16.15 | 29.41 | 21.23 |
| Cases averted, million |  |  |  |  |
| CVD cases | 5.20 | 3.44 | 3.23 | 0.97 |
| CVD deaths | 1.00 | 0.70 | 0.50 | 0.11 |
| Diabetes cases^g^ | 0.20 | 0.13 | 0.11 | 0.02 |
| QALYs gained, million^h^ | 13.4 | 8.98 | 7.41 | 1.86 |
| Change in policy costs, $ billion^i^ |  |  |  |  |
| Administrative costs | 24.1 | 16.3 | 12.7 | 3.5 |
| Food subsidy costs | 392.6 | 262.6 | 219.0 | 58.5 |
| Change in formal healthcare cost, $ billion^j^ | -160.2 | -106.5 | -99.2 | -30.1 |
| Net costs, healthcare perspective, $ billion^k^ | 256.5 | 172.4 | 132.5 | 31.9 |
| ICER, $/QALY^l^ | 19,184 | 19,192 | 17,877 | 17,149 |

^a^ Health outcomes were evaluated among Medicare, Medicaid, and dual-eligible beneficiaries aged 35-80 years at baseline, and followed until death or age 100, whichever came first.

^b^ Includes Medicare only, Medicaid only, and dual-eligible beneficiaries. The number of overall population (n=82 million) is not equal to sum of Medicare (n=58.2 million) and Medicaid (n=35.2 million) because dual-eligible (n=11.4 million) is included in both Medicare and Medicaid.

^c^ Includes Medicare only and dual-eligible beneficiaries.

^d^ Includes Medicaid only and dual-eligible beneficiaries.

^e^ Beneficiaries on both Medicare and Medicaid.

^f^ The average number of years for all simulated individuals in the model.

^g^ We did not identify probable or convincing evidence of etiologic effects of fruits and vegetables on diabetes, the *F&V incentive* resulted in a slightly higher number of diabetes cases compared to a base-case of no new intervention due to increased overall survival from prevented cardiovascular disease (CVD).

^h^ Quality-adjusted life years (QALYs) were discounted at 3% annually.

^i^ Policy costs included total administrative costs and food subsidy costs. All costs were inflated in 2017 dollars.

^j^ Negative costs indicate health-related savings. Formal healthcare costs were calculated from the change in total healthcare costs associated with CVD events including chronic/acute disease states, surgical procedures, screening costs, and drug costs; with diabetes cases including institutional care, outpatient care, outpatient medications and supplies, discounted at 3% annually.

^k^ Net costs from a healthcare perspective = Policy costs – formal healthcare savings, discounted at 3% annually.

^l^ According to the ACC/AHA, ICERs below $50,000/QALY and at $50,000-$150,000/QALY are considered highly cost effective and cost-effective, respectively.[55]

QALY - quality-adjusted life-year, ICER - incremental cost-effectiveness ratio

**Table U**. Lifetime health gains per million persons of 20, 30, and 50% F&V incentive and healthy food incentive programs through Medicare and Medicaid.^a^

|  |  | **Overall^b^** | **Medicare^c^** | **Medicaid^d^** | **Dual-eligible^e^** |
| --- | --- | --- | --- | --- | --- |
| US adults (35-80y) represented, million |  | 82.0 | 58.2 | 35.2 | 11.4 |
| **Scenario 1: F&V incentive** |  |  |  |  |  |
| **20% incentive** |  |  |  |  |  |
| Cases averted per million persons |  |  |  |  |  |
| CVD cases |  | 16,045 | 14,925 | 22,503 | 22,164 |
| CVD deaths |  | 2,948 | 2,963 | 2,863 | 2,174 |
| Diabetes cases^f^ |  | -62 | -30 | -236 | -256 |
| QALYs gained per million persons |  | 38,345 | 37,370 | 47,173 | 37,783 |
| **30% incentive** |  |  |  |  |  |
| Cases averted per million persons |  |  |  |  |  |
| CVD cases |  | 23,830 | 22,174 | 33,147 | 32,483 |
| CVD deaths |  | 4,292 | 4,257 | 4,290 | 3,267 |
| Diabetes cases^f^ |  | -68 | - 57 | -298 | -291 |
| QALYs gained per million persons |  | 57,540 | 54,746 | 69,601 | 55,963 |
| **50% incentive** |  |  |  |  |  |
| Cases averted per million persons, million |  |  |  |  |  |
| CVD cases |  | 38,152 | 35,922 | 52,728 | 51,570 |
| CVD deaths |  | 6,809 | 6,879 | 7,063 | 5,469 |
| Diabetes cases^f^ |  | -133 | -99 | -437 | -369 |
| QALYs gained per million persons |  | 92,426 | 89,144 | 111,790 | 91,236 |
| **Scenario 2: Healthy food incentive** |  |  |  |  |  |
| **20% incentive** |  |  |  |  |  |
| Cases averted, million |  |  |  |  |  |
| CVD cases |  | 27,656 | 25,432 | 40,848 | 37,449 |
| CVD deaths |  | 5,212 | 5,216 | 5,995 | 4,004 |
| Diabetes cases^f^ |  | 1,043 | 924 | 1,247 | 599 |
| QALYs gained per million persons |  | 70,520 | 66,256 | 92,580 | 68,677 |
| **30% incentive** |  |  |  |  |  |
| Cases averted, million |  |  |  |  |  |
| CVD cases |  | 40,427 | 37,335 | 59,261 | 54,523 |
| CVD deaths |  | 7,671 | 7,556 | 8,936 | 6,049 |
| Diabetes cases^f^ |  | 1,521 | 1,383 | 1,879 | 930 |
| QALYs gained per million persons |  | 103,386 | 97,561 | 135,016 | 101,757 |
| **50% incentive** |  |  |  |  |  |
| Cases averted, million |  |  |  |  |  |
| CVD cases |  | 63,421 | 59,119 | 91,813 | 85,417 |
| CVD deaths |  | 12,235 | 11,976 | 14,209 | 10,067 |
| Diabetes cases^f^ |  | 2,403 | 2,217 | 3,079 | 1,666 |
| QALYs gained per million persons |  | 163,066 | 154,395 | 210,528 | 163,303 |

^a^ Health outcomes were evaluated among Medicare, Medicaid, and dual-eligible beneficiaries aged 35-80 years at baseline, and followed until death or age 100, whichever came first.

^b^ Includes Medicare only, Medicaid only, and dual-eligible beneficiaries. The number of overall population (n=82 million) is not equal to sum of Medicare (n=58.2 million) and Medicaid (n=35.2 million) because dual-eligible (n=11.4 million) is included in both Medicare and Medicaid.

^c^ Includes Medicare only and dual-eligible beneficiaries.

^d^ Includes Medicaid only and dual-eligible beneficiaries.

^e^ Beneficiaries on both Medicare and Medicaid.

^f^ We did not identify probable or convincing evidence of etiologic effects of fruits and vegetables on diabetes, the *F&V incentive* resulted in a slightly higher number of diabetes cases compared to a base-case of no new intervention due to increased overall survival from prevented cardiovascular disease (CVD).


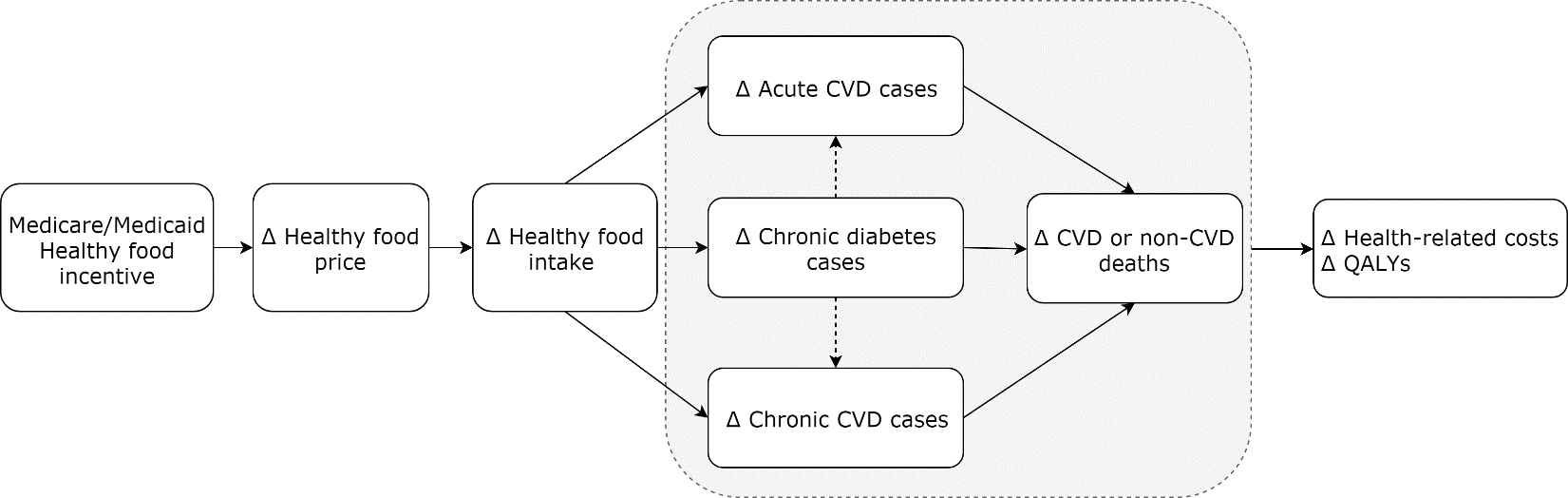


**Fig A.** Logic pathway linking the healthy food incentive program through Medicare and Medicaid to changes in cardiometabolic health outcomes, QALYs, and health-related costs. The model simulates first the life courses of synthetic individuals aged 35-80y under the ‘no new intervention’ scenario and estimates their healthy food intake, incidence of cardiovascular disease (CVD) and diabetes, quality-adjusted life years (QALYs), and event associated health-related costs. Then, it simulates the life courses of the same synthetic individuals under both healthy food incentive scenarios and generates annual estimated changes in each health outcome at the individual level. The dotted lines represent that diabetes was treated as a CVD risk factor to predict acute/chronic CVD cases.

| **A** | **D** |
| --- | --- |
| **B** | **E** |
| **C** | 17,238  16,933  17,842  18,184  **F** |

**Fig B**. Estimated reductions in total CVD events averted (Panel A), diabetes cases averted (Panel B), QALYs (Panel C), healthcare savings (Panel D), net costs (Panel E), and ICERs (Panel F) of the 30% F&V incentive program through Medicare and Medicaid by insurance type over 5, 10, 20 years and lifetime from a healthcare perspective. Numbers indicate the values for lifetime analysis. We did not identify probable or convincing evidence of etiologic effects of fruits and vegetables on diabetes, the F&V incentive resulted in a slightly higher number of diabetes cases due to increased overall survival from prevented CVD. Net costs were calculated policy costs minus healthcare savings. Incremental cost-effectiveness ratios (ICERs) were calculated as the total net change in costs (policy costs minus healthcare savings) divided by the net change in quality-adjusted life years (QALYs).

|  |  |
| --- | --- |
|  |  |
|  |  |

**Fig C**. Estimated health-related savings, net costs, and incremental cost-effectiveness ratio of the 30% F&V incentive and healthy food incentive programs through Medicare and Medicaid by insurance over 5, 10, 20 years and lifetime from a societal perspective. Numbers indicate the values for lifetime analysis. Health-related savings included savings from formal healthcare, informal healthcare, and productivity costs. Net costs were calculated policy costs minus health-related savings. Incremental cost-effectiveness ratios (ICERs) were calculated as the total net change in costs (policy costs minus healthcare savings) divided by the net change in quality-adjusted life years (QALYs).

**Healthy food incentive**

**F&V incentive**

| **D** |  |
| --- | --- |
| **F**  **C** | 70.8  100.2  160.2 |
|  | 57.1  111.1  256.5 |

**E**

**B**

**A**

**G**

**Fig D.** Estimated reductions in total CVD events averted (Panel A), diabetes cases averted (Panel B), quality-adjusted life years (QALYs) (Panel C), healthcare savings (Panel D), subsidy costs (Panel E), net costs (Panel F) and incremental cost-effectiveness ratio (Panel G) of the 20, 30, and 50% healthy food incentive program through Medicare and Medicaid over 5, 10, 20 years and a lifetime. Values are shown from a healthcare perspective. Numbers indicate the values for lifetime analysis. Incremental cost-effectiveness ratios (ICERs) were calculated as the change in net costs (policy costs minus healthcare savings) divided by the net change in quality-adjusted life years (QALYs).

# **References**

1. Neumann PJ, Sanders GD, Russell LB, Siegel JE, Ganiats TG. Cost-effectiveness in health and medicine: Oxford University Press; 2017

2. Gaziano TA, Bertram M, Tollman SM, Hofman KJ. Hypertension education and adherence in South Africa: a cost-effectiveness analysis of community health workers. BMC Public Health. 2014;14:240. Epub 2014/03/13. doi: 10.1186/1471-2458-14-240. PubMed PMID: 24606986; PubMed Central PMCID: PMC3973979.

3. Pandya A, Sy S, Cho S, Weinstein MC, Gaziano TA. Cost-effectiveness of 10-Year Risk Thresholds for Initiation of Statin Therapy for Primary Prevention of Cardiovascular Disease. JAMA. 2015;314(2):142-50. Epub 2015/07/15. doi: 10.1001/jama.2015.6822. PubMed PMID: 26172894; PubMed Central PMCID: PMC4797634.

4. Mozaffarian D, Liu J, Sy S, Huang Y, Rehm C, Lee Y, et al. Cost-effectiveness of financial incentives and disincentives for improving food purchases and health through the US Supplemental Nutrition Assistance Program (SNAP): A microsimulation study. PLoS Med. 2018;15(10):e1002661. Epub 2018/10/03. doi: 10.1371/journal.pmed.1002661. PubMed PMID: 30278053; PubMed Central PMCID: PMC6168180

5. Micha R, Shulkin ML, Penalvo JL, Khatibzadeh S, Singh GM, Rao M, et al. Etiologic effects and optimal intakes of foods and nutrients for risk of cardiovascular diseases and diabetes: Systematic reviews and meta-analyses from the Nutrition and Chronic Diseases Expert Group (NutriCoDE). PLoS One. 2017;12(4):e0175149. Epub 2017/04/28. doi: 10.1371/journal.pone.0175149. PubMed PMID: 28448503; PubMed Central PMCID: PMC5407851.

6. Hu FB, Rimm EB, Stampfer MJ, Ascherio A, Spiegelman D, Willett WC. Prospective study of major dietary patterns and risk of coronary heart disease in men. Am J Clin Nutr. 2000;72(4):912-21. Epub 2000/09/30. doi: 10.1093/ajcn/72.4.912. PubMed PMID: 11010931.

7. Fung TT, Willett WC, Stampfer MJ, Manson JE, Hu FB. Dietary patterns and the risk of coronary heart disease in women. Arch Intern Med. 2001;161(15):1857-62. Epub 2001/08/30. PubMed PMID: 11493127.

8. Fung TT, Rexrode KM, Mantzoros CS, Manson JE, Willett WC, Hu FB. Mediterranean diet and incidence of and mortality from coronary heart disease and stroke in women. Circulation. 2009;119(8):1093-100. PubMed PMID: 19221219.

9. Trichopoulou A, Bamia C, Trichopoulos D. Anatomy of health effects of Mediterranean diet: Greek EPIC prospective cohort study. BMJ. 2009;338:b2337. Epub 2009/06/25. doi: 10.1136/bmj.b2337. PubMed PMID: 19549997; PubMed Central PMCID: PMC3272659.

10. Martinez-Gonzalez MA, de la Fuente-Arrillaga C, Lopez-Del-Burgo C, Vazquez-Ruiz Z, Benito S, Ruiz-Canela M. Low consumption of fruit and vegetables and risk of chronic disease: a review of the epidemiological evidence and temporal trends among Spanish graduates. Public Health Nutr. 2011;14(12a):2309-15. Epub 2011/12/15. doi: 10.1017/s1368980011002564. PubMed PMID: 22166189.

11. Appel LJ, Moore TJ, Obarzanek E, Vollmer WM, Svetkey LP, Sacks FM, et al. A clinical trial of the effects of dietary patterns on blood pressure. DASH Collaborative Research Group. N Engl J Med. 1997;336(16):1117-24. Epub 1997/04/17. doi: 10.1056/nejm199704173361601. PubMed PMID: 9099655.

12. Sacks FM, Svetkey LP, Vollmer WM, Appel LJ, Bray GA, Harsha D, et al. Effects on blood pressure of reduced dietary sodium and the Dietary Approaches to Stop Hypertension (DASH) diet. DASH-Sodium Collaborative Research Group. N Engl J Med. 2001;344(1):3-10. Epub 2001/01/04. doi: 10.1056/nejm200101043440101. PubMed PMID: 11136953.

13. Appel LJ, Sacks FM, Carey VJ, Obarzanek E, Swain JF, Miller ER, 3rd, et al. Effects of protein, monounsaturated fat, and carbohydrate intake on blood pressure and serum lipids: results of the OmniHeart randomized trial. JAMA. 2005;294(19):2455-64. PubMed PMID: 16287956.

14. Di Angelantonio E, Sarwar N, Perry P, Kaptoge S, Ray KK, Thompson A, et al. Major lipids, apolipoproteins, and risk of vascular disease. JAMA. 2009;302(18):1993-2000. Epub 2009/11/12. doi: 10.1001/jama.2009.1619. PubMed PMID: 19903920; PubMed Central PMCID: PMC3284229.

15. Baigent C, Keech A, Kearney PM, Blackwell L, Buck G, Pollicino C, et al. Efficacy and safety of cholesterol-lowering treatment: prospective meta-analysis of data from 90,056 participants in 14 randomised trials of statins. Lancet. 2005;366(9493):1267-78. PubMed PMID: 16214597.

16. Law MR, Wald NJ, Rudnicka AR. Quantifying effect of statins on low density lipoprotein cholesterol, ischaemic heart disease, and stroke: systematic review and meta-analysis. BMJ. 2003;326(7404):1423. Epub 2003/06/28. doi: 10.1136/bmj.326.7404.1423. PubMed PMID: 12829554; PubMed Central PMCID: PMC162260.

17. Lawes CM, Rodgers A, Bennett DA, Parag V, Suh I, Ueshima H, et al. Blood pressure and cardiovascular disease in the Asia Pacific region. J Hypertens. 2003;21(4):707-16. Epub 2003/03/27. doi: 10.1097/01.hjh.0000052492.18130.07. PubMed PMID: 12658016.

18. Lewington S, Clarke R, Qizilbash N, Peto R, Collins R, Prospective Studies C. Age-specific relevance of usual blood pressure to vascular mortality: a meta-analysis of individual data for one million adults in 61 prospective studies. Lancet. 2002;360(9349):1903-13. PubMed PMID: 12493255.

19. Estruch R, Ros E, Salas-Salvado J, Covas MI, Corella D, Aros F, et al. Primary prevention of cardiovascular disease with a Mediterranean diet. N Engl J Med. 2013;368(14):1279-90. Epub 2013/02/26. doi: 10.1056/NEJMoa1200303. PubMed PMID: 23432189.

20. Pandya A, Sy S, Cho S, Alam S, Weinstein MC, Gaziano TA. Validation of a Cardiovascular Disease Policy Microsimulation Model Using Both Survival and Receiver Operating Characteristic Curves. Med Decis Making. 2017;37(7):802-14. Epub 2017/05/12. doi: 10.1177/0272989X17706081. PubMed PMID: 28490271; PubMed Central PMCID: PMC5577377.

21. Sullivan PW, Ghushchyan V. Preference-based EQ-5D index scores for chronic conditions in the United States. Medical Decision Making. 2006;26(4):410-20.

22. Economic Research Service (ERS). U.S. Department of Agriculture (USDA). Supplementary Nutrition Assistance Program Participation and Costs [cited 2018 Dec 19]. Available from: <https://fns-prod.azureedge.net/sites/default/files/pd/SNAPsummary.pdf>

23. Bartlett S KJ, Olsho L, Logan C, Blocklin M, Beauregard M, et al, . Evaluation of the Healthy Incentives Pilot (HIP): final report. Alexandria (VA): US Department of Agriculture Food and Nutrition Service; 2014 [cited 2018 Dec 15]. Available from: <https://fns-prod.azureedge.net/sites/default/files/ops/HIP-Final.pdf>.

24. United States Department of Agriculture. Economic Research Service. Quarterly Food-at-Home Price Database. Washington (DC): US Department of Agriculture Economic Research Service; 2016 [cited 2018 Sep 3]. Available from: <https://www.ers.usda.gov/data-products/quarterly-food-at-home-price-database>.

25. O'Sullivan AK, Rubin J, Nyambose J, Kuznik A, Cohen DJ, Thompson D. Cost estimation of cardiovascular disease events in the US. Pharmacoeconomics. 2011;29(8):693-704. Epub 2011/05/19. doi: 10.2165/11584620-000000000-00000. PubMed PMID: 21585226.

26. Centers for Disease Control and Prevention. National Center for Health Statistics. Overview of NHANES Survey Design and Weights [cited 2018 March 12]. Available from: <https://www.cdc.gov/nchs/tutorials/environmental/orientation/sample_design/>

27. Afshin A, Penalvo JL, Del Gobbo L, Silva J, Michaelson M, O'Flaherty M, et al. The prospective impact of food pricing on improving dietary consumption: A systematic review and meta-analysis. PLoS One. 2017;12(3):e0172277. Epub 2017/03/02. doi: 10.1371/journal.pone.0172277. PubMed PMID: 28249003; PubMed Central PMCID: PMC5332034.

28. Green R, Cornelsen L, Dangour AD, Turner R, Shankar B, Mazzocchi M, et al. The effect of rising food prices on food consumption: systematic review with meta-regression. BMJ. 2013;346:f3703. Epub 2013/06/19. doi: 10.1136/bmj.f3703. PubMed PMID: 23775799; PubMed Central PMCID: PMC3685509.

29. Micha R, Penalvo JL, Cudhea F, Imamura F, Rehm CD, Mozaffarian D. Association Between Dietary Factors and Mortality From Heart Disease, Stroke, and Type 2 Diabetes in the United States. JAMA. 2017;317(9):912-24. Epub 2017/03/08. doi: 10.1001/jama.2017.0947. PubMed PMID: 28267855.

30. Mozaffarian D, Rimm EB. Fish intake, contaminants, and human health: evaluating the risks and the benefits. JAMA. 2006;296(15):1885-99. Epub 2006/10/19. doi: 10.1001/jama.296.15.1885. PubMed PMID: 17047219.

31. He FJ, MacGregor GA. Effect of modest salt reduction on blood pressure: a meta-analysis of randomized trials. Implications for public health. J Hum Hypertens. 2002;16(11):761-70. PubMed PMID: 12444537.

32. Estruch R, Ros E, Salas-Salvado J, Covas MI, Corella D, Aros F, et al. Primary prevention of cardiovascular disease with a Mediterranean diet. N Engl J Med. 2013;368(14):1279-90. Epub 2013/02/26. doi: 10.1056/NEJMoa1200303. PubMed PMID: 23432189.

33. Mozaffarian D. Mediterranean diet for primary prevention of cardiovascular disease. N Engl J Med. 2013;369(7):673-4. Epub 2013/08/16. doi: 10.1056/NEJMc1306659#SA3. PubMed PMID: 23944310.

34. Food and Nutrition Service (FNS), U.S. Department of Agriculture (USDA). SNAP National Level Annual Summary: Participation and Costs, 1969-2016. Nov 2017

35. Centers for Medicaid and Medicare Services. National Health Expenditure Accounts: Methodology Paper, 2014 Definitions, Sources, and Methods 2015.

36. U.S. Department of Agriculture, Economic Research Service. Quarterly Food-at-Home Price Database. Available from: <https://www.ers.usda.gov/data-products/quarterly-food-at-home-price-database/>.

37. Lee KK, Cipriano LE, Owens DK, Go AS, Hlatky MA. Cost-effectiveness of using high-sensitivity C-reactive protein to identify intermediate- and low-cardiovascular-risk individuals for statin therapy. Circulation. 2010;122(15):1478-87. Epub 2010/09/30. doi: 10.1161/circulationaha.110.947960. PubMed PMID: 20876434.

38. Pignone M, Earnshaw S, Tice JA, Pletcher MJ. Aspirin, statins, or both drugs for the primary prevention of coronary heart disease events in men: a cost-utility analysis. Ann Intern Med. 2006;144(5):326-36. Epub 2006/03/08. PubMed PMID: 16520473.

39. Pletcher MJ, Lazar L, Bibbins-Domingo K, Moran A, Rodondi N, Coxson P, et al. Comparing impact and cost-effectiveness of primary prevention strategies for lipid-lowering. Ann Intern Med. 2009;150(4):243-54. Epub 2009/02/18. PubMed PMID: 19221376.

40. Lazar LD, Pletcher MJ, Coxson PG, Bibbins-Domingo K, Goldman L. Cost-effectiveness of statin therapy for primary prevention in a low-cost statin era. Circulation. 2011;124(2):146-53. Epub 2011/06/29. doi: 10.1161/circulationaha.110.986349. PubMed PMID: 21709063.

41. Thomson Corporation. Red book: Pharmacy's Fundamental Reference. Montvale, NJ Thomson PDR; 2009.

42. Nuckols TK, Aledort JE, Adams J, Lai J, Go MH, Keesey J, et al. Cost implications of improving blood pressure management among U.S. adults. Health Serv Res. 2011;46(4):1124-57. Epub 2011/02/11. doi: 10.1111/j.1475-6773.2010.01239.x. PubMed PMID: 21306365; PubMed Central PMCID: PMC3165181.

43. Shah ND, Mason J, Kurt M, Denton BT, Schaefer AJ, Montori VM, et al. Comparative effectiveness of guidelines for the management of hyperlipidemia and hypertension for type 2 diabetes patients. PLoS One. 2011;6(1):e16170. Epub 2011/02/02. doi: 10.1371/journal.pone.0016170. PubMed PMID: 21283569; PubMed Central PMCID: PMC3026790.

44. Economic costs of diabetes in the U.S. in 2012. Diabetes Care. 2013;36(4):1033-46. Epub 2013/03/08. doi: 10.2337/dc12-2625. PubMed PMID: 23468086; PubMed Central PMCID: PMC3609540.

45. Zhuo X, Zhang P, Hoerger TJ. Lifetime direct medical costs of treating type 2 diabetes and diabetic complications. Am J Prev Med. 2013;45(3):253-61. Epub 2013/08/21. doi: 10.1016/j.amepre.2013.04.017. PubMed PMID: 23953350.

46. Russell LB, Ibuka Y, Carr D. How Much Time Do Patients Spend on Outpatient Visits?: The American Time Use Survey. Patient. 2008;1(3):211-22. Epub 2008/07/01. doi: 10.2165/1312067-200801030-00008. PubMed PMID: 22272927.

47. Department of Labor Bureau of Labor Statistics. Table 1300. Age of reference person: Annual expenditure means, shares, standard errors, and coefficients of variation, Consumer Expenditure Survey, 2016. Washington (DC): Department of Labor Bureau of Labor Statistics; 2016 [cited 2018 Sep 3]. Available from: <https://www.bls.gov/cex/2016/combined/age.pdf>.

48. Kim DD, Basu A, Duffy SQ and Zarkin GA. Appendix A. Worked Example 1: Cost-effectiveness of treatments for individuals with alcohol use disorders: A reference case analysis. In: P. Neumann, ed. Cost-effectiveness in Health and Medicine. 2nd Edition. Oxford: Oxford University Press; 2017.

49. U.S. Department of Labor. The Bureau of Labor Statistics' Consumer Price Index 2017 [cited 2017 October 16]. Available from: <https://www.bls.gov/data/#prices>.

50. U.S. Department of Agriculture, Economic Research Service. Quarterly Food-at-Home Price Database. Available from: <https://www.ers.usda.gov/data-products/quarterly-food-at-home-price-database/>.

51. Zhang H, Plutzky J, Skentzos S, Morrison F, Mar P, Shubina M, et al. Discontinuation of statins in routine care settings: a cohort study. Ann Intern Med. 2013;158(7):526-34. Epub 2013/04/03. doi: 10.7326/0003-4819-158-7-201304020-00004. PubMed PMID: 23546564; PubMed Central PMCID: PMC3692286.

52. Alsheikh-Ali AA, Ambrose MS, Kuvin JT, Karas RH. The safety of rosuvastatin as used in common clinical practice: a postmarketing analysis. Circulation. 2005;111(23):3051-7. Epub 2005/05/25. doi: 10.1161/circulationaha.105.555482. PubMed PMID: 15911706.

53. Smith SL, Fischoff R, Klemp T. Medicare RBRVS 2011: The Physician's Guide. Chicago, IL: American Medical Association; 2011.

54. Centers for Disease Control and Prevention (CDC). National Center for Health Statistics (NCHS). National Health and Nutrition Examination Survey [cited 2017 Nov 13]. Available from: <https://wwwn.cdc.gov/nchs/nhanes/default.aspx>.

55. Anderson JL, Heidenreich PA, Barnett PG, Creager MA, Fonarow GC, Gibbons RJ, et al. ACC/AHA statement on cost/value methodology in clinical practice guidelines and performance measures: a report of the American College of Cardiology/American Heart Association Task Force on Performance Measures and Task Force on Practice Guidelines. J Am Coll Cardiol. 2014;63(21):2304-22. Epub 2014/04/01. doi: 10.1016/j.jacc.2014.03.016. PubMed PMID: 24681044.
